# Novel GPR120 Agonists with Improved Pharmacokinetic Profiles for the Treatment of Type 2 Diabetes

**DOI:** 10.3390/molecules26226907

**Published:** 2021-11-16

**Authors:** Guoxia Ji, Qinghua Guo, Qidi Xue, Ruifang Kong, Shiben Wang, Kang Lei, Renmin Liu, Xuekun Wang

**Affiliations:** 1 School of Pharmaceutical Sciences, Liaocheng University, 1 Hunan Street, Liaocheng 252059, China; jiguoxia@lcu.edu.cn (G.J.); guoqinghua712@163.com (Q.G.); qidixue63@gmail.com (Q.X.); a23891436@163.com (R.K.); wangshiben@lcu.edu.cn (S.W.); leikang@lcu.edu.cn (K.L.); 2 School of Chemistry and Chemical Engineering, Liaocheng University, 1 Hunan Street, Liaocheng 252059, China

**Keywords:** GPR120, synthesis, type 2 diabetes, pharmacokinetic profiles

## Abstract

GPR120 is a promising target for the treatment of type 2 diabetes (T2DM), which is activated by free fatty acids (FFAs) and stimulates the release of glucagon-like peptide-1(GLP-1). GLP-1, as an incretin, can enhance glucose-dependent secretion of insulin from pancreatic beta cells and reduce blood glucose. In this study, a series of novel GPR120 agonists were designed and synthesized to improve the stability and hydrophilicity of the phenylpropanoic acid GPR120 agonist TUG-891. Compound **11b** showed excellent GPR120 agonistic activity and pharmacokinetic properties, and could reduce the blood glucose of normal mice in a dose-dependent manner. In addition, no hypoglycemic side effects were observed even at a dose of 100 mg/kg. Moreover, **11b** showed good anti-hyperglycemic effects in diet-induced obese (DIO) mice. Molecular simulation illustrated that compound **11b** could enter the active site of GPR120 and interact with ARG99. Taken together, the results indicate that compound **11b** might be a promising drug candidate for the treatment of T2DM.

## 1. Introduction

Diabetes mellitus accompanied by multiple complications, such as cardiovascular diseases (CVD), nerve damage (neuropathy), kidney damage (nephropathy), and eye disease (leading to retinopathy, visual loss, and even blindness) has become a serious health problem worldwide [1,2]. The main categories of diabetes are type 1 diabetes mellitus (T1DM), type 2 diabetes mellitus (T2DM), and gestational diabetes mellitus. Type 2 diabetes accounts for the vast majority (around 90%) of diabetes worldwide and is mainly characterized by insulin deficiency and insulin resistance, resulting in higher blood glucose levels [1,3]. Although a range of oral antidiabetic agents are available, such as sulfonylureas, metformin, and glitazones, they are unable to achieve satisfactory glycemic control, and are associated with adverse side effects, such as hypoglycemia and cardiovascular outcomes [4,5]. Therefore, oral antidiabetic agents with sustained safety and efficacy are desirable.

G protein-coupled receptors (GPRs) are important signaling molecules involved in many functions. Recently, several studies have reported that GPRS receptors, such as GPR40 [6] and GPR120 [7], play an important role in regulating blood glucose homeostasis. GPR120 (free fatty acid receptor 4, FFAR4) is a 7-transmembrane GPCR that is mainly expressed in the intestine, and other tissues, such as adipose tissue, and macrophages [7]. GPR120 activated by free fatty acids (FFAs), such as omega-3 fatty acids can stimulate the release of glucagon-like peptide-1 (GLP-1), which is a potent incretin hormone that enhances the glucose-dependent secretion of insulin from pancreatic beta cells [8]. Therefore, GPR120 has emerged as an attractive target for the treatment of metabolic diseases such as obesity and T2DM.

**Figure 1:**
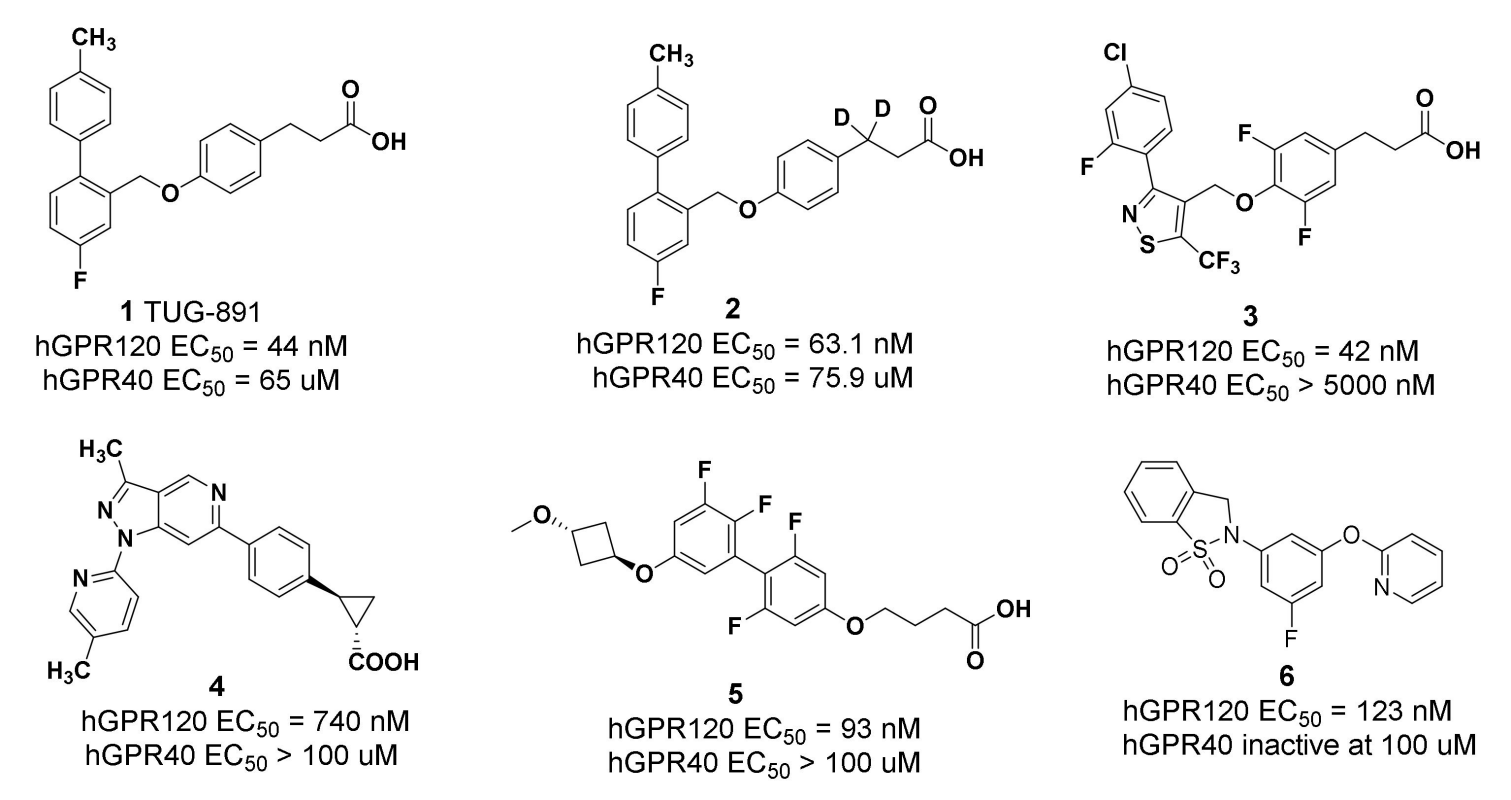
Representative GPR120 agonists.

A few GPR120 agonists containing carboxylic acids or bioelectronic isomers of carboxylic acids have been reported in the literature, and no GPR120 agonists have entered clinical trials (Figure 1) [9,10,11,12,13,14,15]. Compound TUG-891, the first GPR120 agonist with high selectivity and activity, derived from the GPR40 agonist GW9508, is widely used as a pharmacological tool to explore the physiological function of GPR120 [16,17]. However, TUG-891 shows poor metabolic stability and high lipophilicity (*c*log*P* = 5.88), since phenylpropanoic acid is vulnerable to β-oxidation, similar to GPR120 agonists with a phenylpropanoic acid moiety reported previously [18,19] and due to a biphenyl structure with high lipophilicity, which limits the use of TUG-891.

**Figure 2:**
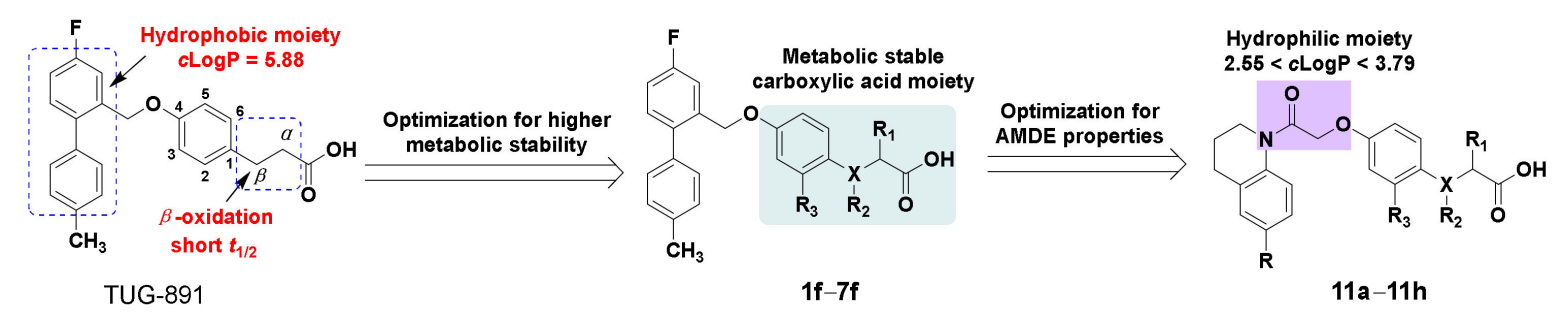
Strategies to improve metabolic stability and hydrophilicity in this study.

In this study, we designed compounds **1f**–**7f** by introducing substituents on the benzene ring, introducing substituents on the α or β position of phenylpropionic acid or substituting oxygen atoms for carbon atoms at the β position of phenylpropionic acid to improve the metabolic stability of TUG-891 [20,21]. Compounds with excellent GPR120 agonist activity and selectivity were used as lead compounds to design compounds **11a**–**11h** for the improvement of hydrophilicity by replacing the biphenyl structure with polar linking groups (Figure 2). Compounds **11a**–**11h** were synthesized and tested for GPR120 activation in vitro. Compounds with excellent GPR120 agonistic activity were selected to evaluate selectivity, pharmacokinetic properties, and antidiabetic activity in vivo. The results showed that compound **11b** had good GPR120 agonistic activity, selectivity, and pharmacokinetic properties, and could reduce the blood glucose of normal mice in a dose-dependent manner. In addition, it showed good anti-hyperglycemic activity in diet-induced obese (DIO) mice. Compound **11b** may be a promising drug candidate for the treatment of T2DM.

## 2. Results and Discussion

### 2.1. Chemistry

**Scheme 1:**
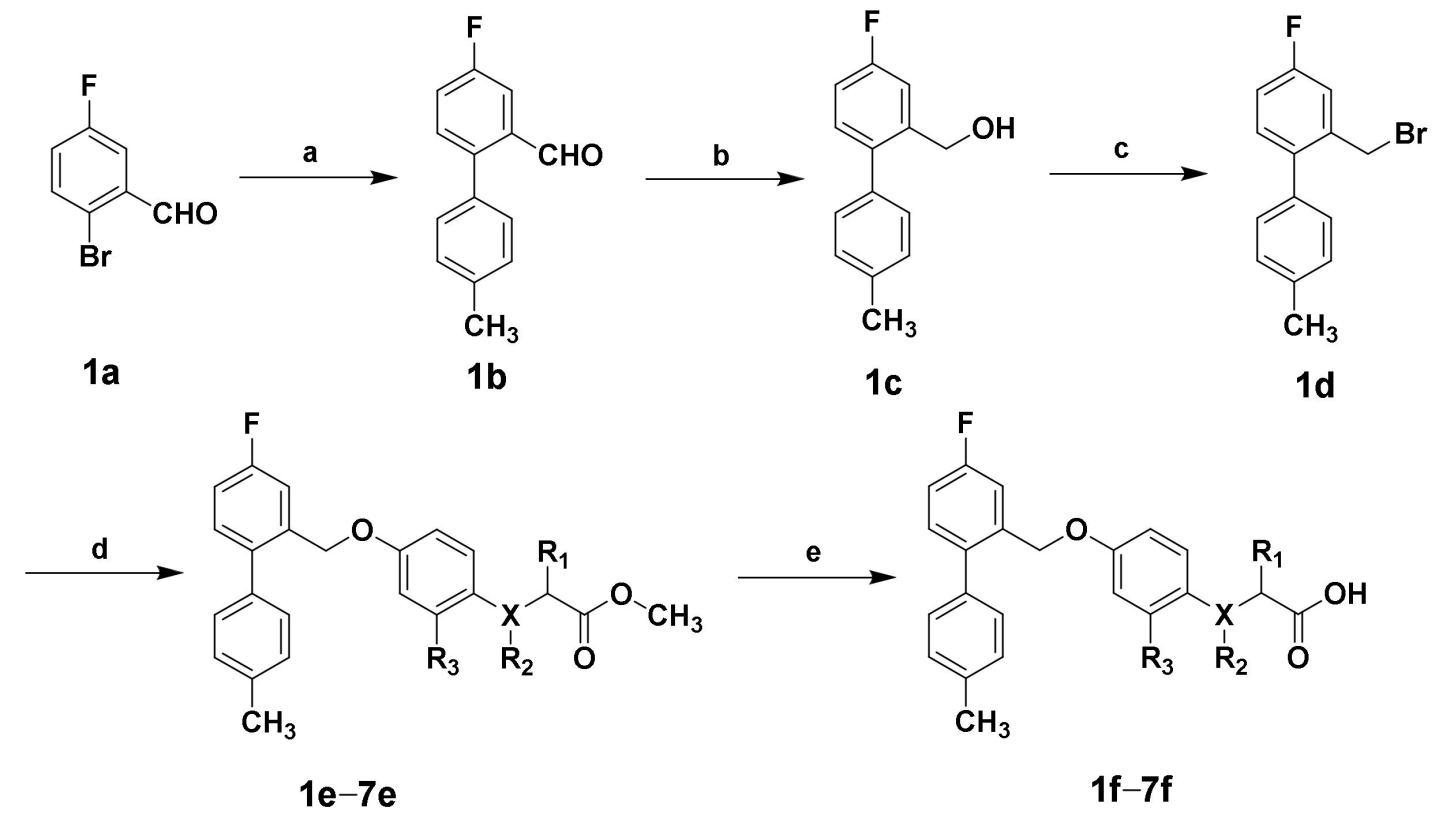
Reagents and conditions: Synthesis of target compounds **1f**–**7f**. (**a**) The 4-tolylboronic acid, Pd(PPh_3_)_4_, Na_2_CO_3_, toluene, ethanol, H_2_O, 80 °C, overnight, 78.2%; (**b**) NaBH_4_, CH_3_OH, THF, 0 °C, 1 h; (**c**) PBr_3_, CH_2_Cl_2_, 0 °C, 1 h, 53.1% of two steps; (**d**) suitable phenolic derivative, K_2_CO_3_, DMF, room temperature, 12 h; (**e**) NaOH, H_2_O, CH_3_OH, THF, 2 h, 53.4%–60.7% of two steps.

The target compounds **1f**–**7f** were obtained according to the synthetic route summarized in Scheme 1. The 2-bromo-5-fluorobenzaldehyde and 4-tolylboronic acids were used as starting materials to synthesize intermediate **1b** by the Suzuki-Miyaura cross-coupling reaction in the catalysis of Pd(PPh_3_)_4_. Intermediate **1c** was synthesized by reducing intermediate **1b** with sodium borohydride, and was converted to **1d** by substituting hydroxyl with phosphorus tribromide. Condensation of **1d** with appropriate phenolic hydroxyl by the Williamson ether synthesis reaction afforded intermediate **1e**–**7e**, followed by hydrolysis with sodium hydroxide to generate the target compounds **1f**–**7f**. The structures of the target compounds were confirmed by ^1^H-NMR, ^13^C-NMR, and HRMS.

**Scheme 2:**
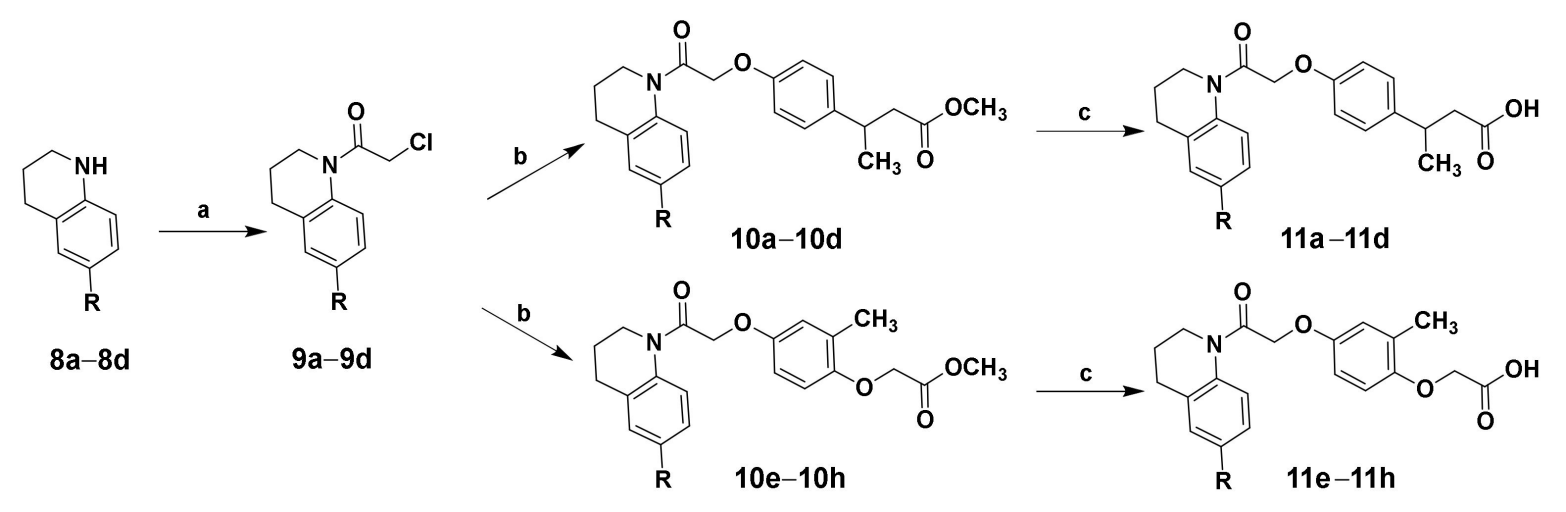
Reagents and conditions: Synthesis of target compounds **11a**–**11h**. (**a**) Chloroacetyl chloride, TEA, DCM, 0 °C, overnight, 77.4%; (**b**) methyl 3-(4-hydroxyphenyl)butanoate or methyl 2-(4-hydroxy-2-methylphenoxy)acetate, K_2_CO_3_, DMF, room temperature, 12 h; (**c**) NaOH, H_2_O, CH_3_OH, THF, 2 h, 50.8%–58.9% of two steps.

Compound **2f** with *β*-methylphenylpropionic acid and compound **6f** with 2-methylphenoxyacetic acid exhibited excellent GPR120 agonist activity and selectivity in vitro screening. Based on the structures of compounds **2f** and **6f**, compounds **11a**–**11h** were designed to improve hydrophilicity by replacing the biphenyl structure with polar linking groups. The target compounds **11a**–**11h** were obtained according to the synthetic route summarized in Scheme 2. Unsubstituted or substituted 1,2,3,4-tetrahydroquinoline was used to synthesize chloride intermediate **9a**–**9d** by acylation of the nitrogen atom with chloroacetyl chloride. Intermediate **10a**–**10h** were synthesized by condensing chloride intermediate **9a**–**9h** and methyl 3-(4-hydroxyphenyl)butanoate or methyl 2-(4-hydroxy-2-methylphenoxy)acetate. They were hydrolyzed with sodium hydroxide to generate the target compounds **11a**–**11h**. The structures of the target compounds were confirmed by ^1^H-NMR, ^13^C-NMR, and HRMS.

### 2.2. Pharmacology

#### 2.2.1. GPR120 Agonistic Activity and Selectivity

The GPR120 agonistic activities of the target compounds **1f**–**7f** and **11a**–**11h** were investigated by monitoring the Ca^2+^ signal, and TUG-891 was used as a positive control. The results showed that the introduction of methyl at the α position of phenylpropionic acid decreased the GPR120 agonist activity compared with TUG-891, and the introduction of methyl at the β position of phenylpropionic acid increased the GPR120 agonist activity (**2f** > TUG-891 > **1f**). This indicated that the steric hindrance increased after the introduction of methyl at the α position, which affected the interaction between carboxylic acid and GPR120 receptor. The introduction of a substituent at the ortho position of phenylpropionic acid decreased the GPR120 agonistic activity, regardless of whether it was an electron-withdrawing or electron-donating group (**3f** < **4f** < TUG-891). The phenoxyacetic acid derivative **5f** obtained by substituting an oxygen atom for the β-position carbon atom of phenylpropionic acid could also retain the GPR120 agonistic activity. The introduction of methyl into the ortho position of **5f** improved the GPR120 agonistic activity, whereas the introduction of fluorine atoms decreased it (**7f** < **5f** < **6f**). Compounds **2f** and **6f**, with excellent GPR120 agonistic activity, were tested for GPR40 agonistic activity, and the results showed that the EC_50_ of the compounds **2f** and **6f** to GPR40 were greater than 50 μM, which indicated that compounds **2f** and **6f** exhibited excellent selectivity (Table 1).

Although compounds **2f** and **6f** showed excellent GPR120 agonistic activity and selectivity, their lipophilicity was too high to conform to Lipinski’s “Rule of Five” (Log*P* ≤ 5.0; *c*Log*P*(**2f**) = 6.21; *c*Log*P*(**6f**) = 5.59), which affected the pharmacokinetic properties in vivo. To improve the hydrophilicity, compounds **11a**–**11h** (2.55 < *c*Log*P* < 3.79) were designed and synthesized by replacing the biphenyl structure of compounds **2f** or **6f** with a polar linking group. The results indicated that compounds **11a**–**11h** still exhibited the GPR120 agonistic activity. The GPR120 agonistic activity of compound **11a** without substituents on the tetrahydroquinoline ring was reduced compared with compound **2f**, and the introduction of electron-donating groups, such as methyl and methoxy groups, at the 6-position of tetrahydroquinoline ring enhanced the GPR120 agonistic activity compared with compound **11a**. However, the introduction of electron-withdrawing groups decreased it (**11d** < **11a** < **11c** < **11b**). The GPR120 agonistic activity of **11e** was about one-quarter of **6f**. However, the GPR120 agonistic activity was increased after the introduction of an electron-withdrawing group or an electron-donating group at the 6 position of the tetrahydroquinoline ring, and the electron-donating group was more beneficial in increasing the activity (**11e** < **11f** < **11h** < **11g**). The selectivity of compounds **11b** and **11g** with excellent GPR120 agonistic activity was evaluated, and the results showed that compounds **11b** and **11g** exhibited excellent selectivity (Table 2). Therefore, the potent agonists **11b** and **11g**, with excellent selectivity and potency on GPR120 were selected for further investigation.

#### 2.2.2. Pharmacokinetic Evaluation of Compounds **11b** and **11g** in C57BL/6 Mice

The original purpose of this study was to improve the metabolic stability of TUG-891 in vivo. The pharmacokinetic profiles of **11b** and **11g** were obtained from C57BL/6 mice. The results showed that both compounds **11b** and **11g** reached *C*_max_ after 30 min of gavage administration at a dose of 10 mg/kg, and the *C*_max_ of **11b** (*C*_max_ = 2530 ng/mL) was higher than the positive control TUG-891 (*C*_max_ = 2160 ng/mL), while *C*_max_ of compound **11g** decreased slightly (*C*_max_ = 1846 ng/mL). Compounds **11b** and **11g** exhibited longer half-lives and higher maximum plasma concentrations than TUG-891, which indicated that compounds **11b** and **11g** possessed excellent metabolic stability in vivo (Table 3).

#### 2.2.3. Oral Glucose Tolerance Test of Compound **11b** in Normal ICR Mice

**Figure 3:**
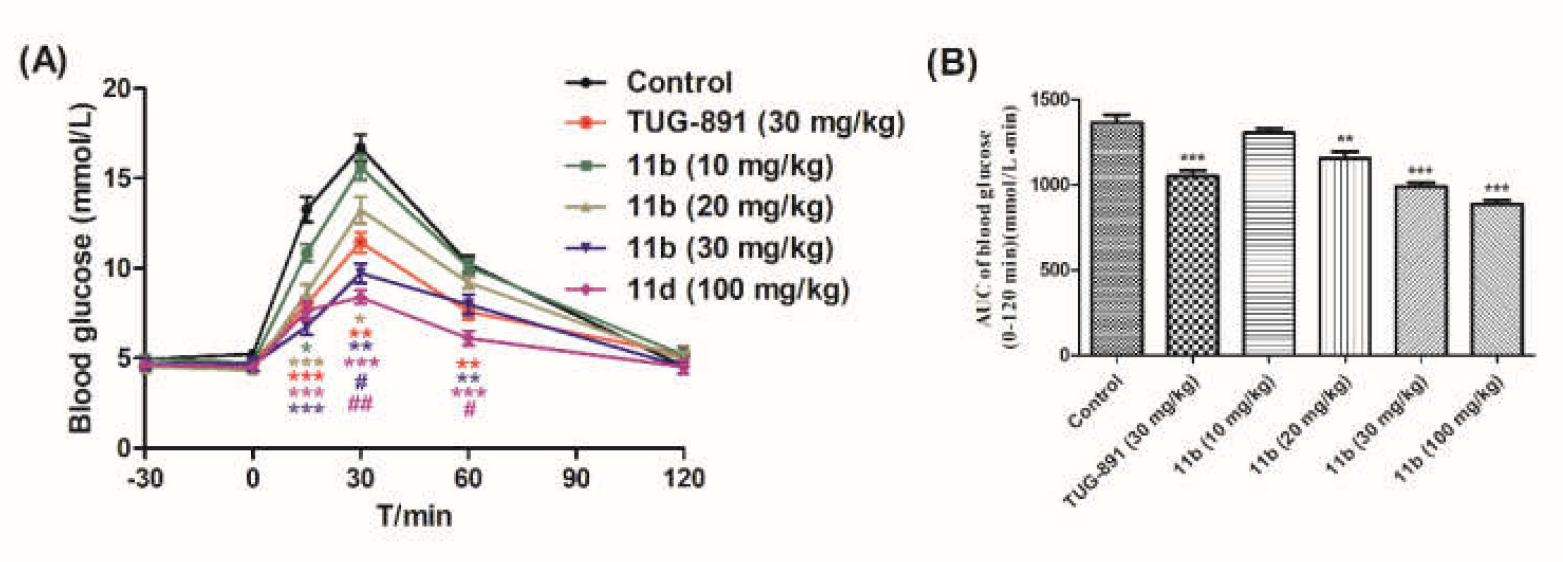
Effects of compound **11b** and TUG-891 on plasma glucose levels during the oral glucose tolerance tests (oGTT) in fasting ICR mice. (**A**) Time-dependent changes of blood glucose levels after oral administration of compound **11b**, followed by the oral glucose load (3 g/kg). (**B**) AUC_-30–120 min_ of plasma glucose levels. Values are expressed as the mean ± SEM (*n* = 6). * *p* ≤ 0.05 compared with vehicle-treated ICR mice by Student’s *t* test; ** *p* ≤ 0.01 compared with vehicle-treated ICR mice by Student’s *t* test; *** *p* ≤ 0.001 compared with vehicle-treated ICR mice by Student’s *t* test; ^#^ *p* ≤ 0.05 compared with TUG-891-treated ICR mice by Student’s *t* test; ^##^ *p* ≤ 0.01 compared with TUG-891-treated ICR mice by Student’s *t* test.

Based on the experimental results, compound **11b** with excellent activity, high selectivity, and metabolic stability was selected for acute efficacy evaluation in ICR mice by oral glucose tolerance tests (oGTTs). ICR mice that were fasted overnight were dosed compound **11b** by gavage administration at doses of 10, 20, 30, and 100 mg/kg 30 min before the oral glucose challenge at a 3.0 g/kg dose. TUG-891 was used as a control, and glucose levels were measured from 30 min pre-glucose to 120 min post-glucose administration. The results showed that the blood glucose level increased after glucose administration, reached the maximum concentration at 30 min, and returned to normal levels at 120 min. Compound **11b** lowered the blood glucose levels in a dose-dependent manner, and significantly reduced blood glucose at a dose of 20 mg/kg. The anti-hyperglycemic effects of **11b** were better than TUG-891 at 30 min after the oral glucose challenge at the dose of 30 mg/kg. In addition, no hypoglycemia was observed even at a dose of 100 mg/kg during the experiment, which indicated that compound **11b** had no risk of hypoglycemia (Figure 3A,B).

#### 2.2.4. Anti-Hyperglycemic Effects of Compound **11b** Explored in DIO Mice

**Figure 4:**
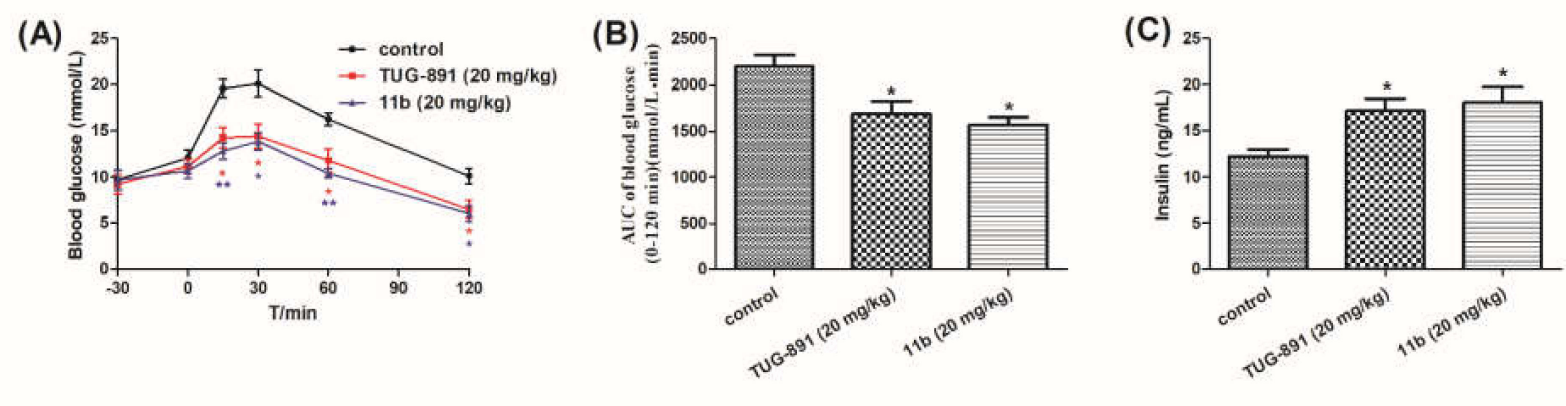
Effects of compound **11b** and TUG-891 on plasma glucose levels during oGTT in fasting DIO mice. (**A**) The changes of blood glucose levels after oral administration of compound **11b**, followed by the oral glucose load (2 g/kg); (**B**) AUC_-30–120 min_ of plasma glucose levels. (**C**) Insulin levels at 30 min after the glucose load. Values are expressed as the mean ± SEM (*n* = 6). * *p* ≤ 0.05 compared with vehicle-treated DIO mice by Student’s *t* test; ** *p* ≤ 0.01 compared with vehicle-treated DIO mice by Student’s *t* test.

Diet-induced obese (DIO) mice were obtained by feeding male C57BL/6 mice with high-fat diet for 8 weeks. There was hardly any glucose elimination in DIO mice, which suggests severe glucose intolerance [22,23]. The anti-hyperglycemic effects of compound **11b** were evaluated in DIO mice to assess the consequences of GPR120 agonists in an acute model of type 2 diabetes. The results showed that the blood glucose levels of the groups increased after glucose administration, and reached the maximum concentration at 30 min. Compound **11b** showed anti-diabetic effects equivalent to TUG-891, and the blood glucose levels reached normal levels at 120 min (Figure 4A,B). The insulin levels at 30 min after oral administration of glucose were tested, and the results showed that compound **11b** and TUG-891 significantly increased insulin levels (Figure 4C).

#### 2.2.5. Molecular Modeling

**Figure 5:**
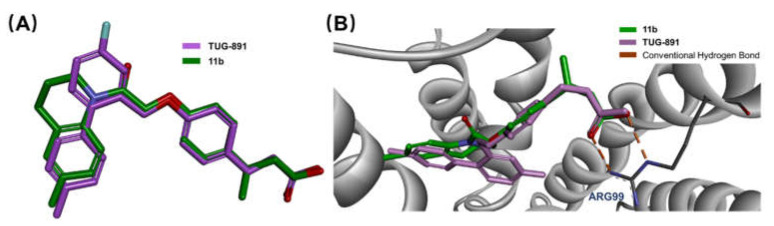
(**A**) The conformation overlap between TUG-891 and compound **11b**. (**B**) Docking of compound **11b** and TUG-891 inside of a homology GPR120 model.

Molecular overlay studies were performed to simulate the difference in molecular conformation between compound **11b** and TUG-891. Compound **11b** with a hydrophilic linking group exhibited an excellent degree of overlap with TUG-891 (Figure 5A). The crystal structure of the GPR120 receptor has not been reported. Homology modeling was performed with Modeler in Accelrys Discovery Studio 2020 using the crystal structure of Turkey β1 adrenoceptor 20 (PDB code 6IBL) [24], neurotensin receptor21 (PDB code 4XES) [25], and β2-adrenoceptor (PDB code 3P0G) [26] as templates. The structure with the lowest discrete optimized protein energy (DOPE) score was used for the molecular docking analysis. The molecular docking model showed that compound **11b** was engaged in conventional hydrogen bond interactions with Arg99, which plays a very important role in GPR120 activation [16] (Figure 5B). All of the simulation results suggested that compound **11b** exerted anti-diabetic activity and was related to the GPR120 receptor.

## 3. Materials and Methods

### 3.1. Chemistry

All of the commercially available materials and reagents were used without purification unless otherwise indicated. Purification by column chromatography was performed using silica gel (200–300 mesh). The melting points of the target compounds **1f**–**7f** and **11a**–**11h** were determined using an x-5 micro melting point apparatus, which was uncorrected. The NMR spectra (500 MHz for ^1^H-NMR and 125 MHz for ^13^C-NMR spectra) were recorded using a Bruker AVANCE NEO 500 instrument (Bruker, Germany, compounds were dissolved in DMSO*_d6_*). Chemical shifts are shown as values relative to the internal standard (tetramethylsilane), and coupling constants (*J* values) are given in hertz (Hz). High-resolution mass spectrometry was conducted using an UPLC G2-XS Qtof spectrometer (Waters, Milford, MA, USA). The names of the compounds were obtained by ChemBioDraw Ultra 14.0 (Cambridge Soft, Cambridge, MA, USA). The NMR and HRMS spectra of compounds **1f**–**7f** and **11a**–**11h** are presented in Supplementary Materials Figures S1–S45.

#### 3.1.1. 4-Fluoro-4′-methyl-[1,1′-biphenyl]-2-carbaldehyde (**1b**)

The 2-bromo-5-fluorobenzaldehyde (2.00 g, 9.85 mmol) and 4-tolylboronic acids (1.34 g, 9.85 mmol) were dissolved in a mixture of 1N sodium carbonate aq. (20 mL), ethanol (10 mL), and toluene (20 mL). After nitrogen substitution, Pd(PPh_3_)_4_ (0.57 g, 0.49 mmol) was added as a catalyst. The reaction mixture was stirred at 80 °C under nitrogen atmosphere for 12 h. After the reaction was complete (TLC examination), the reaction mixture was cooled, and diluted with ethyl acetate (50 mL). The insoluble material of the mixture was filtered off through Celite. The organic layer of the filtrate was washed with water and brine, dried over anhydrous sodium sulfate, and concentrated under reduced pressure. The residue was purified by silica gel column chromatography using a mixture of petroleum ether/ethyl acetate (30:1, *v*/*v*) as eluent to afford the desired product **1b** (1.65 g, 78.2%) as a solid. ^1^H-NMR (500 MHz, DMSO-*d*_6_) δ 9.83 (d, *J* = 3.2 Hz, 1H), 7.64–7.58 (m, 2H), 7.58–7.54 (m, 1H), 7.33 (s, 4H), 2.39 (s, 3H).

#### 3.1.2. (4-Fluoro-4′-methyl-[1,1′-biphenyl]-2-yl)methanol (**1c**)

To a solution of **1b** (1.50 g, 7.00 mmol) in THF (20 mL) and MeOH (15 mL), sodium borohydride was added portion wise (0.13 g, 3.5 mmol) at 0 °C. The mixture was stirred at 0 °C for 30 min and quenched with dilute hydrochloric acid (pH = 3) after the reaction was complete (TLC examination). The mixture was extracted with ethyl acetate (3 × 20 mL), and the organic fractions were combined, washed with water (2 × 30 mL), and saturated brine (2 × 30 mL) prior to drying over anhydrous sodium sulfate. After filtration and concentration using a rotary evaporator under reduced pressure to afford a residue of 1.42 g, the residue was used in the next step without further purification. 1H-NMR (500 MHz, DMSO-*d_6_*) δ 7.34 (dd, *J* = 10.4, 2.9 Hz, 1H), 7.26–7.19 (m, 5H), 7.12 (td, *J* = 8.5, 2.9 Hz, 1H), 5.37–5.26 (m, 1H), 4.40 (d, *J* = 5.3 Hz, 2H), 2.35 (s, 3H).

#### 3.1.3. 2-(Bromomethyl)-4-fluoro-4′-methyl-1,1′-biphenyl (**1d**)

To a solution of the obtained **1c** (1.42 g, 6.57 mmol) in dichloromethane (30 mL), phosphorus tribromide was slowly added (0.89 g, 3.28 mmol) and dissolved in dichloromethane (5 mL) at 0 °C. After stirring at 0 °C for 1 h, the reaction mixture was quenched with cold water (20 mL) and stirred for another 2 h at room temperature. The mixture was extracted with dichloromethane (3 × 20 mL), and the organic fractions were combined, washed with water (2 × 30 mL), and saturated brine (2 × 30 mL) prior to drying over anhydrous sodium sulfate. After filtration and concentration using a rotary evaporator under reduced pressure, the residue was purified by silica gel column chromatography using a mixture of petroleum ether/ethyl acetate (50:1, *v*/*v*) as eluent to afford a colorless solid (1.36 g, 53.1% of two steps). ^1^H-NMR (500 MHz, DMSO-*d*_6_) δ 7.34 (dd, *J* = 10.4, 2.9 Hz, 1H), 7.26–7.19 (m, 5H), 7.12 (td, *J* = 8.5, 2.9 Hz, 1H), 4.39 (s, 2H), 2.35 (s, 3H).

#### 3.1.4. General Synthetic Procedure for the Target Compounds **1f**–**7f**

To a solution of **1d** (0.20 g, 0.72 mmol, 1.0 equiv) and phenol derivatives (1.0 equiv) in acetone, K_2_CO_3_ was added (0.20 g, 1.43 mmol, 2.0 equiv) at room temperature. The reaction mixture was stirred at 60 °C for 12 h, and the insoluble material of the mixture was filtered after the reaction was complete (TLC examination). The filtrate was evaporated under reduced pressure, and the residue **1e**–**7e** was used in the next step without further purification.

To a solution of **1e**–**7e** (0.2 g, 1.0 equiv) in THF (10 mL), CH_3_OH (5 mL), and H_2_O (5 mL), NaOH was added (2 N, 2.0 equiv) at room temperature. The reaction mixture was stirred for 2 h and acidified with HCl (1 N) after the hydrolysis was complete (TLC examination). The mixture was extracted with ethyl acetate (3 × 20 mL), and the combined organic phases were washed with brine (2 × 30 mL), dried over anhydrous sodium sulfate, and filtered. The filtrate was evaporated under a vacuum and the residue was purified by column chromatography (MeOH/CH_2_Cl_2_, 20:1, *v*/*v*) to give the appropriate target compounds **1f**–**7f**. The yields, melting points, and spectral data (^1^H-NMR, ^13^C-NMR, and HRMS) of compounds **1f**–**7f** are given below.

*3-(4-((4-Fluoro-4′-methyl-[1,1′-biphenyl]-2-yl)methoxy)phenyl)-2-methylpropanoic acid* (**1f**): Colorless solid, yield 53.4% of two steps; m.p. 103~104 °C; ^1^H-NMR (500 MHz, DMSO-*d*_6_) δ 12.11 (s, 1H), 7.41 (dd, *J* = 9.9, 2.7 Hz, 2H), 7.38–7.32 (m, 1H), 7.31–7.22 (m, 5H), 7.07 (d, *J* = 8.5 Hz, 2H), 6.79 (d, *J* = 8.5 Hz, 2H), 4.90 (s, 2H), 2.81 (d, *J* = 6.4 Hz, 1H), 2.38–2.30 (m, 2H), 1.08–0.97 (m, 3H); ^13^C-NMR (125 MHz, DMSO-*d*_6_) δ 177.30, 162.83, 160.89, 156.85, 138.01, 137.26, 137.10, 136.47, 132.45, 132.32, 130.36, 129.45, 129.36, 115.98, 115.39, 114.90, 67.54, 41.22, 38.55, 21.15, 17.06; HRMS calcd for C_24_H_23_FO_3_ [M − H]^−^ 377.1553, found 377.1556.

*3-(4-((4-Fluoro-4′-methyl-[1,1′-biphenyl]-2-yl)methoxy)phenyl)butanoic acid* (**2f**): Colorless solid, yield 57.2% of two steps; m.p. 100~101 °C; ^1^H-NMR (500 MHz, DMSO-*d*_6_) δ 12.03 (s, 1H), 7.40 (dd, *J* = 9.9, 2.7 Hz, 2H), 7.38–7.32 (m, 1H), 7.32–7.22 (m, 5H), 7.13 (d, *J* = 8.6 Hz, 2H), 6.79 (d, *J* = 8.7 Hz, 2H), 4.90 (s, 2H), 3.15–3.03 (m, 1H), 2.44 (d, *J* = 7.5 Hz, 2H), 2.34 (s, 3H), 1.16 (d, *J* = 6.9 Hz, 3H); ^13^C-NMR (126 MHz, DMSO-*d*_6_) δ 173.68, 162.84, 160.90, 156.80, 139.00, 137.98, 137.27, 137.14, 136.47, 132.33, 129.47, 129.35, 128.18, 115.94, 115.38, 114.98, 67.55, 42.85, 35.51, 22.55, 21.16; HRMS calcd for C_24_H_23_FO_3_ [M − H]^−^ 377.1553, found 377.1558.

*3-(4-((4-Fluoro-4′-methyl-[1,1′-biphenyl]-2-yl)methoxy)-2-methylphenyl)propanoic acid* (**3f**): Colorless solid, yield 55.4% of two steps; m.p. 116~117 °C; ^1^H-NMR (500 MHz, DMSO-*d*_6_) δ 12.16 (s, 1H), 7.39 (dd, *J* = 9.9, 2.8 Hz, 1H), 7.34 (dd, *J* = 8.6, 5.9 Hz, 1H), 7.29 (d, *J* = 7.9 Hz, 2H), 7.26–7.22 (m, 2H), 7.00 (d, *J* = 8.3 Hz, 1H), 6.69 (s, 1H), 6.59 (dd, *J* = 8.3, 2.7 Hz, 1H), 4.89 (s, 2H), 2.72 (t, *J* = 7.8 Hz, 2H), 2.43 (t, *J* = 7.7 Hz, 2H), 2.34 (s, 3H), 2.16 (s, 3H); ^13^C-NMR (125 MHz, DMSO-*d*_6_) δ 174.27, 162.82, 160.88, 156.67, 140.67, 138.04, 137.34, 136.51, 132.31, 131.22, 129.45, 129.37, 128.46, 116.01, 115.64, 115.36, 112.31, 67.46, 34.38, 28.31, 21.15, 18.42; HRMS calcd for C_24_H_23_FO_3_ [M − H]^−^ 377.1553, found 377.1556.

*3-(2-Fluoro-4-((4-fluoro-4′-methyl-[1,1′-biphenyl]-2-yl)methoxy)phenyl)propanoic acid* (**4f**): Colorless solid, yield 60.7% of two steps; m.p. 101~102 °C; ^1^H-NMR (500 MHz, DMSO-*d*_6_) δ 12.21 (s, 1H), 7.42 (dd, *J* = 9.9, 2.7 Hz, 1H), 7.35 (dd, *J* = 8.5, 5.9 Hz, 1H), 7.31–7.20 (m, 5H), 7.03 (t, *J* = 9.3 Hz, 1H), 6.84 (dd, *J* = 6.2, 3.1 Hz, 2H), 6.76–6.69 (m, 1H), 4.89 (s, 2H), 2.77 (t, *J* = 7.7 Hz, 3H), 2.50–2.46 (m, 2H), 2.34 (s, 3H); ^13^C-NMR (125 MHz, DMSO-*d*_6_) δ 173.89, 162.81, 160.87, 156.45, 154.55, 138.14, 137.27, 136.85, 136.48, 132.37, 129.41, 128.78, 117.22, 116.12, 115.51, 113.87, 68.14, 34.12, 24.29, 21.15; HRMS calcd for C_23_H_20_F_2_O_3_ [M − H]^−^ 381.1302, found 381.1304.

*2-(4-((4-Fluoro-4′-methyl-[1,1′-biphenyl]-2-yl)methoxy)phenoxy)acetic acid* (**5f**): Colorless solid, yield 54.9% of two steps; m.p. 119~120 °C; ^1^H-NMR (500 MHz, DMSO-*d*_6_) δ 7.41 (dd, *J* = 9.9, 2.7 Hz, 1H), 7.34 (dd, *J* = 8.5, 5.9 Hz, 1H), 7.31–7.19 (m, 5H), 6.81 (s, 4H), 4.87 (s, 2H), 4.58 (s, 2H), 2.34 (s, 3H); ^13^C-NMR (125 MHz, DMSO-*d*_6_) δ 170.85, 162.82, 160.89, 152.76, 152.64, 138.00, 137.26, 137.18, 136.49, 132.30, 129.45, 129.34, 115.94, 115.35, 68.11, 65.60, 21.15; HRMS calcd for C_22_H_19_FO_4_ [M − H]^−^ 365.1189, found 365.1188.

*2-(4-((4-Fluoro-4′-methyl-[1,1′-biphenyl]-2-yl)methoxy)-2-methylphenoxy)acetic acid* (**6f**): Colorless solid, yield 53.8% of two steps; m.p. 115~116 °C; ^1^H-NMR (500 MHz, DMSO-*d*_6_) δ 7.39 (dd, *J* = 10.0, 2.8 Hz, 1H), 7.34 (dd, *J* = 8.5, 5.9 Hz, 1H), 7.32–7.21 (m, 5H), 6.75–6.67 (m, 2H), 6.60 (dd, *J* = 8.9, 3.1 Hz, 1H), 4.85 (s, 2H), 4.59 (s, 2H), 2.34 (s, 3H), 2.13 (s, 3H); ^13^C-NMR (125 MHz, DMSO-*d*_6_) δ 170.98, 162.83, 160.89, 152.39, 150.93, 138.00, 137.33, 137.26, 136.51, 132.28, 129.45, 129.36, 127.76, 118.02, 115.96, 115.33, 112.79, 112.44, 67.95, 65.94, 21.15, 16.61; HRMS calcd for C_23_H_21_FO_4_ [M − H]^−^ 379.1346, found 379.1351.

*2-(2-Fluoro-4-((4-fluoro-4′-methyl-[1,1′-biphenyl]-2-yl)methoxy)phenoxy)acetic acid* (**7f**): Colorless solid, yield 56.8% of two steps; m.p. 109~110 °C; ^1^H-NMR (500 MHz, DMSO-*d*_6_) δ 7.42 (dd, *J* = 9.9, 2.7 Hz, 1H), 7.35 (dd, *J* = 8.5, 5.8 Hz, 1H), 7.31–7.20 (m, 5H), 6.97 (t, *J* = 9.4 Hz, 1H), 6.62 (d, *J* = 8.6 Hz, 1H), 4.88 (s, 2H), 4.66 (s, 2H), 2.33 (s, 3H); ^13^C-NMR (125 MHz, DMSO-*d*_6_) δ 170.64, 162.80, 160.86, 153.15, 152.65, 151.21, 138.18, 137.28, 136.69, 136.43, 132.35, 129.45, 129.34, 116.30, 116.19, 116.13, 115.54, 110.34, 104.50, 68.36, 66.77, 21.14; HRMS calcd for C_22_H_18_F_2_O_4_ [M − H]^−^ 383.1095, found 383.1110.

#### 3.1.5. General Synthetic Procedure for Intermediates **9a**–**9d**

To a solution of unsubstituted or substituted 1,2,3,4-tetrahydroquinoline (1.00 g, 1.0 equiv) and trimethylamine (2.0 equiv) in CH_2_Cl_2_ (20 mL), chloroacetyl chloride was added slowly and dissolved in CH_2_Cl_2_ (10 mL) at 0 °C. The reaction mixture was stirred overnight at room temperature and water (30 mL) was added to quench the reaction after TLC examination, indicating that the reaction was complete. The mixture was extracted with CH_2_Cl_2_ (3 × 20 mL), and the combined organic phases were washed with brine (2 × 50 mL), dried over anhydrous sodium sulfate, and filtered. The filtrate was evaporated under a vacuum and the residue was purified by column chromatography (petroleum ether/ethyl acetate 10:1, *v*/*v*) to give the intermediates **9a**–**9d**.

#### 3.1.6. General Synthetic Procedure for Target Compounds **11a**–**11h**

To a solution of **9a**–**9d** (0.20 g, 1.0 equiv) and methyl 3-(4-hydroxyphenyl)butanoate or methyl 2-(4-hydroxy-2-methylphenoxy)acetate (1.0 equiv) in acetone, K_2_CO_3_ was added (2.0 equiv) at room temperature. The reaction mixture was stirred at 60 °C for 12 h, and the insoluble material of the mixture was filtered after the reaction was complete. The filtrate was evaporated under reduced pressure, and the residue **10a**–**10h** was used in the next step without further purification.

To a solution of **10a**–**10h** (0.2 g, 1.0 equiv) in THF (10 mL), CH_3_OH (5 mL), and H_2_O (5 mL), NaOH was added (2 N, 2.0 equiv) at room temperature. The reaction mixture was stirred for 2 h and acidified with HCl (1 N) after the hydrolysis was complete. The mixture was extracted with ethyl acetate (3 × 20 mL), and the combined organic phases were washed with brine (2 × 30 mL), dried over anhydrous sodium sulfate, and filtered. The filtrate was evaporated under a vacuum and the residue was purified by column chromatography (MeOH/CH_2_Cl_2_, 30:1, *v*/*v*) to give the appropriate target compounds **11a**–**11h**. The yields, melting points, and spectral data (^1^H-NMR, ^13^C-NMR, and HRMS) of compounds **11a**–**11h** are given below.

*3-(4-(2-(3,4-Dihydroquinolin-1(2H)-yl)-2-oxoethoxy)phenyl)butanoic acid* (**11a**): Colorless solid, yield 50.8% of two steps; m.p. 117~118 °C; ^1^H-NMR (500 MHz, DMSO-*d*_6_) δ 7.56 (s, 1H), 7.20 (d, *J* = 7.4 Hz, 1H), 7.18–7.08 (m, 3H), 6.79–6.67 (m, 4H), 4.90 (s, 2H), 3.69 (t, *J* = 6.3 Hz, 2H), 3.14–3.03 (m, 1H), 2.69 (t, *J* = 6.8 Hz, 2H), 2.44 (d, *J* = 7.5 Hz, 2H), 1.92–1.82 (m, 2H), 1.17 (d, *J* = 7.0 Hz, 3H); ^13^C-NMR (125 MHz, DMSO-*d*_6_) δ 173.71, 167.53, 156.67, 138.96, 138.22, 129.11, 128.01, 124.35, 114.75, 66.85, 42.91, 35.52, 26.60, 23.73, 22.61; HRMS calcd for C_21_H_23_NO_4_ [M − H]^−^ 352.1549, found 352.1552.

*3-(4-(2-(6-Methyl-3,4-dihydroquinolin-1(2H)-yl)-2-oxoethoxy)phenyl)butanoic acid* (**11b**): Colorless solid, yield 48.7% of two steps; m.p. 115~116 °C; ^1^H-NMR (500 MHz, DMSO-*d*_6_) δ 11.99 (s, 1H), 7.43 (s, 1H), 7.13 (d, *J* = 8.3 Hz, 2H), 7.01 (s, 1H), 6.97 (d, *J* = 8.4 Hz, 1H), 6.79–6.63 (m, 2H), 4.87 (s, 2H), 3.67 (t, *J* = 6.3 Hz, 2H), 3.15–3.03 (m, 1H), 2.66 (t, *J* = 6.5 Hz, 2H), 2.44 (d, *J* = 7.5 Hz, 2H), 2.26 (s, 3H), 1.91–1.82 (m, 2H), 1.17 (d, *J* = 7.0 Hz, 3H); ^13^C-NMR (125 MHz, DMSO-*d*_6_) δ 173.68, 167.32, 156.72, 138.90, 135.73, 129.54, 128.01, 126.87, 124.14, 114.74, 66.76, 42.85, 35.51, 26.57, 25.60, 23.74, 22.60, 20.90; HRMS calcd for C_22_H_25_NO_4_ [M − H]^−^ 366.1705, found 366.1709.

*3-(4-(2-(6-Methoxy-3,4-dihydroquinolin-1(2H)-yl)-2-oxoethoxy)phenyl)butanoic acid* (**11c**): Colorless solid, yield 51.2% of two steps; m.p. 141~142 °C; ^1^H-NMR (500 MHz, DMSO-*d*_6_) δ 12.00 (s, 1H), 7.42 (s, 1H), 7.13 (d, *J* = 8.1 Hz, 2H), 6.98–6.54 (m, 4H), 4.85 (s, 2H), 3.73 (s, 3H), 3.66 (t, *J* = 6.3 Hz, 2H), 3.14–3.01 (m, 1H), 2.68 (s, 2H), 2.44 (d, *J* = 7.5 Hz, 2H), 1.86 (s, 2H), 1.17 (d, *J* = 7.0 Hz, 3H); ^13^C-NMR (125 MHz, DMSO-*d*_6_) δ 173.69, 167.16, 156.73, 138.90, 131.32, 128.01, 125.46, 114.74, 111.99, 66.70, 55.66, 42.86, 35.51, 26.82, 23.70, 22.60; HRMS calcd for C_22_H_25_NO_5_ [M − H]^−^ 382.1654, found 382.1658.

*3-(4-(2-(6-Fluoro-3,4-dihydroquinolin-1(2H)-yl)-2-oxoethoxy)phenyl)butanoic acid* (**11d**): Colorless solid, yield 53.6% of two steps; m.p. 124~130 °C; ^1^H-NMR (500 MHz, DMSO-*d*_6_) δ 7.63 (s, 1H), 7.13 (d, *J* = 8.3 Hz, 2H), 7.09–6.96 (m, 2H), 6.75 (s, 2H), 6.68–6.64 (m, 1H), 4.90 (s, 2H), 3.68 (t, *J* = 6.2 Hz, 2H), 3.14–3.05 (m, 1H), 2.69 (s, 2H), 2.43 (dd, *J* = 15.5, 7.5 Hz, 2H), 1.93–1.79 (m, 2H), 1.17 (d, *J* = 6.9 Hz, 3H); ^13^C-NMR (125 MHz, DMSO-*d*_6_) δ 173.72, 167.53, 156.01, 138.99, 136.71, 134.55, 127.99 (d, *J* = 5.0 Hz), 126.21, 115.48, 115.38, 115.21, 114.78, 66.85, 43.13, 42.91, 35.52, 26.65, 23.39, 22.63; HRMS calcd for C_21_H_22_FNO_4_ [M − H]^−^ 370.1455, found 370.1454.

*2-(4-(2-(3,4-Dihydroquinolin-1(2H)-yl)-2-oxoethoxy)-2-methylphenoxy)acetic acid* (**11e**): Colorless solid, yield 55.5% of two steps; m.p. 138~139 °C; ^1^H-NMR (500 MHz, DMSO-*d*_6_) δ 12.90 (s, 1H), 7.55 (s, 1H), 7.20 (d, *J* = 7.4 Hz, 1H), 7.16 (td, *J* = 7.7, 1.7 Hz, 1H), 7.10 (t, *J* = 7.4 Hz, 1H), 6.72 (d, *J* = 8.9 Hz, 1H), 6.66 (s, 1H), 6.58 (s, 1H), 4.84 (s, 2H), 4.59 (s, 2H), 3.69 (t, *J* = 6.3 Hz, 2H), 2.70 (t, *J* = 6.6 Hz, 2H), 2.14 (s, 3H), 1.94–1.82 (m, 2H); ^13^C-NMR (125 MHz, DMSO-*d*_6_) δ 170.94, 167.63, 152.32, 150.84, 138.29, 129.11, 127.62, 126.31, 125.33, 124.35, 117.73, 112.75, 112.20, 67.28, 65.86, 43.55, 26.62, 23.75, 16.64; HRMS calcd for C_20_H_21_NO_5_ [M − H]^−^ 354.1341, found 354.1345.

*2-(4-(2-(6-Fluoro-3,4-dihydroquinolin-1(2H)-yl)-2-oxoethoxy)-2-methylphenoxy)acetic acid* (**11f**): Colorless solid, yield 58.9% of two steps; m.p. 152~153 °C; ^1^H-NMR (500 MHz, DMSO-*d*_6_) δ 12.90 (s, 1H), 7.61 (s, 1H), 7.06 (d, *J* = 7.4 Hz, 1H), 6.99 (td, *J* = 8.8, 3.0 Hz, 1H), 6.72 (d, *J* = 8.9 Hz, 1H), 6.67 (s, 1H), 6.61 (s, 1H), 4.84 (s, 2H), 4.59 (s, 2H), 3.67 (t, *J* = 6.2 Hz, 2H), 2.77–2.65 (m, 2H), 2.15 (s, 3H), 1.93–1.82 (m, 2H); ^13^C-NMR (125 MHz, DMSO-*d*_6_) δ 170.94, 167.62, 152.29, 150.86, 134.60, 127.62, 126.20, 117.77, 115.29, 112.74, 112.22, 67.28, 65.87, 26.67, 23.42, 16.64; HRMS calcd for C_20_H_20_FNO_5_ [M − H]^−^ 372.1247, found 372.1245.

*2-(2-Methyl-4-(2-(6-methyl-3,4-dihydroquinolin-1(2H)-yl)-2-oxoethoxy)phenoxy)acetic acid* (**11g**): Colorless solid, yield 56.4% of two steps; m.p. 147~148 °C; ^1^H-NMR (500 MHz, DMSO-*d*_6_) δ 12.89 (s, 1H), 7.42 (s, 1H), 7.01 (s, 1H), 6.97 (d, *J* = 8.4 Hz, 1H), 6.71 (d, *J* = 8.9 Hz, 1H), 6.65 (s, 1H), 6.57 (s, 1H), 4.81 (s, 2H), 4.59 (s, 2H), 3.66 (t, *J* = 6.3 Hz, 2H), 2.66 (t, *J* = 6.6 Hz, 2H), 2.26 (s, 3H), 2.14 (s, 3H), 1.91–1.81 (m, 2H); ^13^C-NMR (125 MHz, DMSO-*d*_6_) δ 170.94, 167.43, 152.36, 150.82, 135.78, 129.53, 127.61, 126.89, 124.13, 117.71, 112.75, 112.19, 67.20, 65.86, 26.58, 23.76, 20.90, 16.64; HRMS calcd for C_21_H_23_NO_5_ [M − H]^−^ 368.1498, found 368.1501.

*2-(4-(2-(6-Methoxy-3,4-dihydroquinolin-1(2H)-yl)-2-oxoethoxy)-2-methylphenoxy)acetic acid* (**11h**): Colorless solid, yield 53.1% of two steps; m.p. 141~142 °C; ^1^H-NMR (500 MHz, DMSO-*d*_6_) δ 12.89 (s, 1H), 7.40 (s, 1H), 6.79 (s, 1H), 6.72 (dd, *J* = 13.5, 9.1 Hz, 2H), 6.66–6.48 (m, 2H), 4.79 (s, 2H), 4.58 (s, 2H), 3.73 (s, 3H), 3.65 (t, *J* = 6.3 Hz, 2H), 2.68 (s, 2H), 2.14 (s, 3H), 1.86 (s, 2H); ^13^C-NMR (125 MHz, DMSO-*d*_6_) δ 170.97, 152.36, 150.84, 131.37, 127.59, 117.72, 112.75, 112.19, 112.02, 65.90, 55.67, 26.83, 23.71, 16.65; HRMS calcd for C_21_H_23_NO_6_ [M − H]^−^ 384.1447, found 384.1447.

### 3.2. Pharmacology

#### 3.2.1. Ca^2+^ Influx Activity of Chinese Hamster Ovary Cells Expressing Human GPR120

Chinese hamster ovary (CHO) cells stably expressing human GPR120 were suspended in growth media at a concentration of 1 × 10^6^ per mL. Then, 20 uL of the cell suspension was added to each well in the 96-well plate (2 × 10^4^ cells/well) and the cells were incubated at 37 °C in a 5% CO_2_ incubator for 24 h. The cell plate was removed from the incubator, the medium was gently discarded, and the wells were washed with 100 μL of Hank’s balanced salt solution (HBSS) per well. The cells were then incubated in HBSS containing fluorescent calcium indicator Fluo-4 AM (2.5 μg/mL) and probenecid (2.5 mmol/L) for 90 min. After removing the Fluo-4 AM and probenecid solution, the wells were washed with HBSS (3 × 100 μL per well), and incubated in HBSS containing probenecid (2.5 mmol/L) for 10 min at 37 °C. The test compounds at various concentrations were added to the cells, and intracellular Ca^2+^ concentrations were measured using FlexStation3 Molecular Devices. The GPR120 agonistic activities of the test compounds were expressed as A/B (increase in the intracellular Ca^2+^ concentration (A) in the test compound-treated cells and (B) in vehicle-treated cells). The EC_50_ value of each compound was calculated using Prism software (version 5.0; GraphPad Software, San Diego, CA, USA). The Calcium influx assay of target compounds on hGPR40-expressing CHO cells was similar to hGPR120.

#### 3.2.2. Animals

Male ICR mice aged 8 weeks, and male C57BL/6 mice aged 8 weeks were purchased from Jinan Pengyue Experimental Animal Breeding Co., Ltd. (Jinan, China) and housed in cages under a 12 h light/dark cycle from 7:00 to 19:00 at controlled temperatures (25–26 °C) and relative humidity (50 ± 10%) throughout the experimental period. Male ICR mice were used to determine the anti-hyperglycemic activity of compound **11b** by oGTTs, and male C57BL/6 mice were used to determine the in vivo pharmacokinetic properties and anti-hyperglycemic effects. TUG-891 was used as a positive control. All of the animals were allowed to eat and drink freely, unless otherwise stated, and were allowed to acclimatize for 1 week before the experiment. All of the animal experimental protocols were performed in accordance with the applicable institutional and governmental regulations concerning the ethical use of animals.

##### Pharmacokinetic Analysis of Compounds **11b** and **11g** in C57BL/6 Mice

The pharmacokinetic properties of compounds **11b**, **11g**, and TUG-891 were determined in C57BL/6 mice. Male C57BL/6 mice weighing 28–32 g were starved for 12 h and randomly divided into three groups (four mice per group). Compounds **11b**, **11g**, and TUG-891 were dissolved in 0.5% methylcellulose (0.5% MC) at a concentration of 10 mg/mL and gavage administered a volume of 1 mL/kg. Blood samples were collected from the retro-orbital plexus into EDTA-containing microcentrifuge tubes at 5, 15, and 30 min and 1, 2, 4, 6, 8, 12, and 24 h after gavage administration, and plasma was separated by centrifugation at 5.645 g for 10 min. Plasma was collected with a pipette and the proteins in the plasma were precipitated with two volumes of acetonitrile containing an internal standard. This was followed by centrifugation at 15.680 g for 10 min after vortexing for 5 min. The supernatant was diluted with acetonitrile and 10 μL of supernatant was analyzed by Waters LC-PDA-MS/MS to determine plasma drug levels. Pharmacokinetic parameters were determined using the mean data from four mice at each time point. Statistical analysis of the data was performed using the DAS 2.1.1 statistical software program (BioVoice, Shanghai, China).

##### Oral Glucose Tolerance Test of Compound **11b** in Normal ICR Mice

Normal male ICR mice were used for oral glucose tolerance tests of compound **11b**, and TUG-891 was used as a positive control. Male ICR starved overnight (12 h) were weighed and randomly divided into six groups (eight mice per group). Compound **11b** at different concentrations and TUG-891 were dissolved in 0.5% MC. Mice were gavage administered a single dose of vehicle (0.5% MC aqueous solution), TUG-891 (30 mg/kg) or compound **11b** (10, 20, 30, and 100 mg/kg), 30 min before the oral glucose loading (3 g/kg). Blood samples were collected via the tail tip before drug administration (-30 min), before glucose loading (time 0), and 15, 30, 60, and 120 min after glucose loading. Blood glucose levels were measured using blood glucose test strips (Sannuo GA-3 type, Changsha, China).

##### Anti-Hyperglycemic Effects of Compound **11b** Explored in DIO Mice

After 1 week of adaptation, male C57BL/6 mice weighing 18–22 g, were fed a high-fat diet (45% calories from fat, from Mediscience Ltd., Yangzhou, China) *ad libitum* for an additional 8 weeks to induce insulin resistance. The mice were used as an acute model of type 2 diabetes to evaluate the anti-hyperglycemic effects of compound **11b**. The DIO mice were starved overnight (12 h), weighed, and randomly divided into the groups (six mice per group). Thereafter, the DIO mice were dosed with a single dose of vehicle (0.5% MC), TUG-891(suspended in vehicle; 20 mg/kg) or **11b** (suspended in vehicle; 20 mg/kg) by gavage administration, 30 min before the oral glucose load (2 g/kg). Blood samples were collected and measured in accordance with the oral glucose tolerance test in normal ICR mice.

### 3.3. Molecular Modeling

Molecular overlay, homologous modeling, and molecular docking were performed using DS2020 (BIOVIA, Waltham, MA, USA) according to the manufacturer’s instructions. The amino sequence of GPR120 was obtained from the UniProtKB database (identifier: Q5NUL3). Similar sequences were identified using the NCBI BLAST. In addition, Turkey β1 adrenoceptor20 (PDB code 6IBL), neurotensin receptor21 (PDB code 4XES), and β2-adrenoceptor (PDB code 3P0G) with high homology were selected as templates to construct a homology model of the GPR120 receptor. The model with the lowest DOPE score was added to the CHARMm force field, and used for molecular docking after energy optimization and evaluation. The structures of the ligands were etched using ChemBioDraw Ultra 14.0. The CDOCKER molecular docking module in DS 2020 was used for molecular docking research on compound **11b**. The docking results were analyzed using the Discovery Studio software.

## 4. Conclusions

A series of novel GPR120 agonists **1f**–**7f** and **11a**–**11h** were designed, synthesized, and evaluated for their anti-hyperglycemic activity in vitro and in vivo. Compounds **1f**–**7f** were designed to improve the metabolic stability of TUG-891 in vivo by introducing substituents on the benzene ring, introducing substituents on the α or β position of phenylpropionic acid or substituting oxygen atoms for carbon atoms at the β position of phenylpropionic acid. Compounds **11a**–**11h** were designed to improve the hydrophilicity by introducing hydrophilic groups. The chemical structures of these compounds were determined by ^1^H-NMR, ^13^C-NMR spectroscopy, and HRMS. The results of pharmacological experiments showed that most of the target compounds exhibited GPR120 agonistic activity. Among them, compound **11b** showed excellent GPR120 agonistic activity and pharmacokinetic properties, and could reduce the blood glucose of normal mice in a dose-dependent manner. In addition, no hypoglycemia side effects were found even at a dose of 100 mg/kg. Moreover, **11b** showed good anti-hyperglycemic effects in DIO mice. Molecular simulation illustrated that compound **11b** could enter the active site of GPR120 and interact with ARG99. Taken together, these results showed that compound **11b** might be a promising drug candidate for the treatment of T2DM.

## Figures and Tables

**Table 1:**
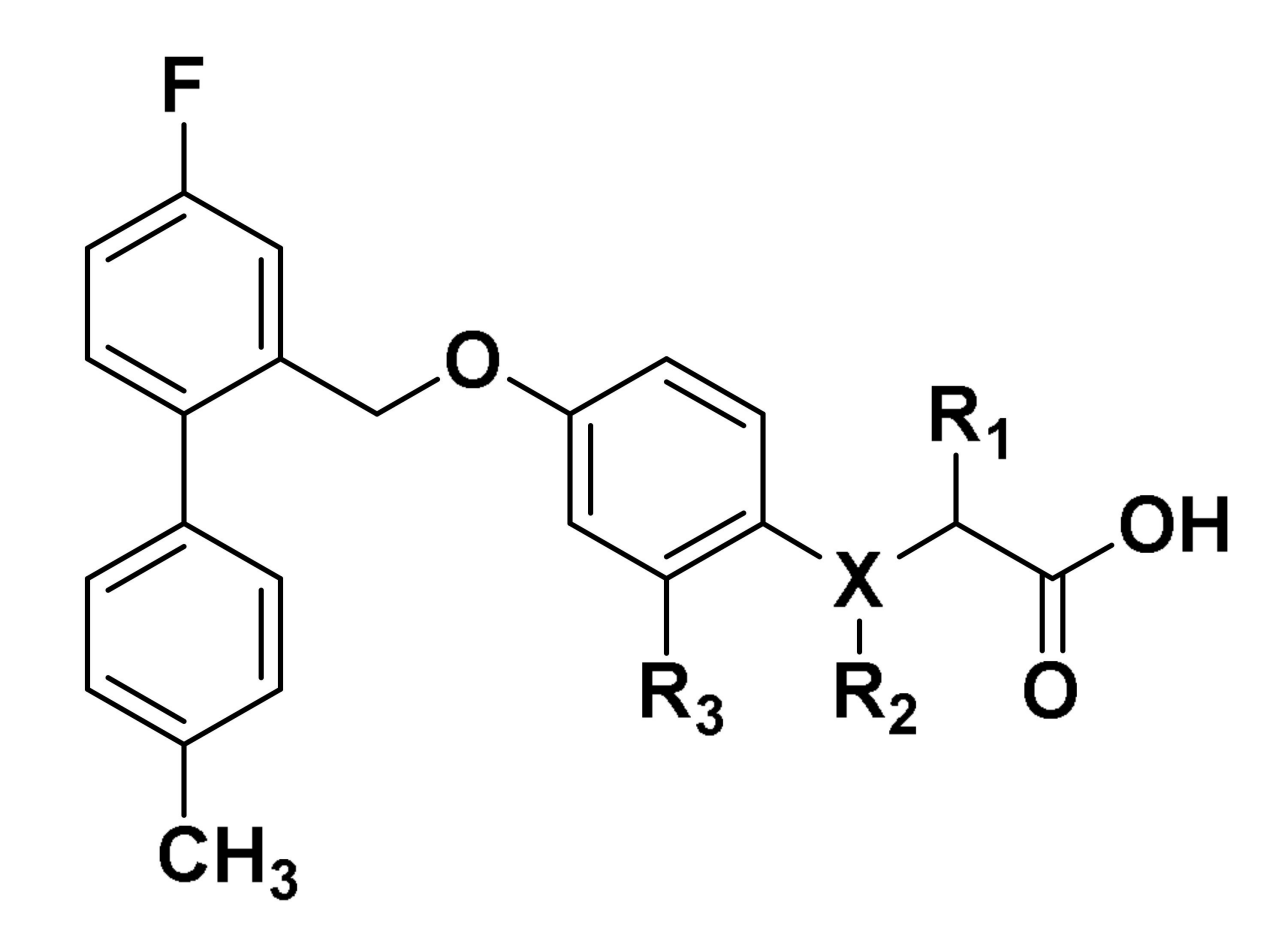
In vitro activities and selectivity of target compounds **1f**–**7f**.

Compound	R_1_	R_2_	R_3_	X	*c*log*P*^a^	GPR120 (EC_50_, nM) ^b^	GPR40 (EC_50_, μM) ^c^	Selectivity
TUG-891	-	-	-	-	5.88	61.7	56.3	912
**1f**	CH_3_	H	H	C	6.45	146.1	ND	ND
**2f**	H	CH_3_	H	C	6.21	52.8	72.5	1373
**3f**	H	H	CH_3_	C	6.37	90.2	ND	ND
**4f**	H	H	F	C	6.04	110.3	ND	ND
**5f**	H	H	H	O	5.10	102.7	ND	ND
**6f**	H	H	CH_3_	O	5.59	72.7	58.9	810
**7f**	H	H	F	O	5.26	106.2	ND	ND

ND: Not determined. ^a^ The *c*log*P* values were calculated by BioByte’s algorithm as implemented in ChemBioDraw Ultra 14.0. ^b^ EC_50_ values for GPR120 activities represent the mean of three determinations. ^c^ EC_50_ values for GPR40 activities represent the mean of three determinations.

**Table 2:**
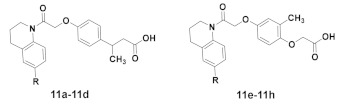
In vitro activities and selectivity of target compounds **11a**–**11h**.

Compound	R	n	*c*log*P*^a^	GPR120 (EC_50_, nM) ^b^	GPR40 (EC_50_, μM) ^c^	Selectivity
TUG-891	-	-		61.7	56.3	912
**11a**	H	2	3.30	112.5	ND	ND
**11b**	CH_3_	2	3.79	71.2	82.5	1159
**11c**	CH_3_O	2	3.18	99.6	ND	ND
**11d**	F	2	3.46	187.4	ND	ND
**11e**	H	2	2.68	254.7	ND	ND
**11f**	6-F	2	2.84	203.6	ND	ND
**11g**	CH_3_	2	3.17	83.4	86.4	1036
**11h**	CH_3_O	2	2.55	145.6	ND	ND

ND: Not determined. ^a^ The *c*log*P* values were calculated by BioByte’s algorithm as implemented in ChemBioDraw Ultra 14.0. ^b^ EC_50_ values for GPR120 activities represent the mean of three determinations. ^c^ EC_50_ values for GPR40 activities represent the mean of three determinations.

**Table 3:** Pharmacokinetic profiles of compound **11b**, **11g**, and TUG-891 in C57BL/6 mice ^a^.

Compound	Dose (mg/kg)	*C*_max_ (ng/mL)	*T*_max_ (min)	AUC (ng·h/mL)	*t*_1/2_ (h)
TUG-891	10	2160 ± 93	15	1675 ± 108	0.66 ± 0.23
**11b**	10	2530 ± 115 *	30	9049 ± 435 ***	1.78 ± 0.42 **
**11g**	10	1846 ± 102	30	6711 ± 467 ***	1.52 ± 0.37 **

^a^ Values are expressed as the mean ± SEM of four C57BL/6 mice. TUG-891 and compounds **11b** and **11g** were suspended in 0.5% MC aqueous solution. * *p* ≤ 0.05 compared with TUG-891-treated by Student’s *t* test; ** *p* ≤ 0.01 compared with TUG-891-treated by Student’s *t* test; *** *p* ≤ 0.001 compared with TUG-891-treated by Student’s *t* test.

## Data Availability

The data presented in this study are available on request from the corresponding author.

## Supplementary Materials

The following are available online. Figures S1–S45: Copies of NMR and HRMS spectra of compounds **1f**–**7f** and **11a**–**11h**.

## Sample Availability

Samples of compounds **1f**–**7f** and **11a**–**11h** are available from the authors.

## References

[1] (2019). IDF Diabetes Atlas.

[2] Cole J.B., Florez J.C. (2020). Genetics of diabetes mellitus and diabetes complications. Nat. Rev. Nephrol..

[3] Zheng Y., Ley S.H., Hu F.B. (2018). Global aetiology and epidemiology of type 2 diabetes mellitus and its complications. Nat. Rev. Endocrinol..

[4] Pappachan J.M., Fernandez C.J., Chacko E.C. (2019). Diabesity and antidiabetic drugs. Mol. Asp. Med..

[5] Eurich D.T., McAlister F.A., Blackburn D.F., Majumdar S.R., Tsuyuki R.T., Varney J., Johnson J.A. (2007). Benefits and harms of antidiabetic agents in patients with diabetes and heart failure: Systematic review. BMJ.

[6] Itoh Y., Kawamata Y., Harada M. (2003). Free fatty acids regulate insulin secretion from pancreatic b cells through GPR40. Nature.

[7] Hirasawa A., Tsumaya K., Awaji T., Katsuma S., Adachi T., Yamada M., Sugimoto Y., Miyazaki S., Tsujimoto G. (2005). Free fatty acids regulate gut incretin glucagon-like peptide-1 secretion through GPR120. Nat. Med..

[8] Müller T.D., Finan B., Bloom S.R., D’Alessio D., Drucker D.J., Flatt P.R., Fritsche A., Gribble F., Grill H.J., Habener J.F. (2019). Glucagon-like peptide 1 (GLP-1). Mol. Metab..

[9] Shimpukade B., Hudson B.D., Hovgaard C.K., Milligan G., Ulven T. (2012). Discovery of a potent and selective GPR120 agonist. J. Med. Chem..

[10] Li Z., Xu X., Li G., Fu X., Liu Y., Feng Y., Wang M., Ouyang Y., Han J. (2017). Improving metabolic stability with deuterium: The discovery of GPU-028, a potent free fatty acid receptor 4 agonists. Bioorg. Med. Chem..

[11] Zhang X., Cai C., Winters M., Wells M., Wall M., Lanter J., Sui Z., Ma J., Novack A., Nashashibi I. (2017). Design, synthesis and SAR of a novel series of heterocyclic phenylpropanoic acids as GPR120 agonists. Bioorg. Med. Chem. Lett..

[12] Zhang X., Cai C., Sui Z., Macielag M., Wang Y., Yan W., Suckow A., Hua H., Bell A., Haug P. (2017). Discovery of an isothiazole-based phenylpropanoic acid GPR120 agonist as a development candidate for type 2 diabetes. ACS Med. Chem. Lett..

[13] McCoull W., Bailey A., Barton P., Birch A.M., Brown A.J., Butler H.S., Boyd S., Butlin R.J., Chappell B., Clarkson P. (2017). Indazole-6-phenylcyclopropylcarboxylic acids as selective GPR120 agonists with in vivo efficacy. J. Med. Chem..

[14] Adams G.L., Velazquez F., Jayne C., Shah U., Miao S., Ashley E.R., Madeira M., Akiyama T.E., Di Salvo J., Suzuki T. (2017). Discovery of chromane propionic acid analogues as selective agonists of GPR120 with in vivo activity in rodents. ACS Med. Chem. Lett..

[15] Azevedo C.M., Watterson K.R., Wargent E.T., Hansen S.V., Hudson B.D., Kepczynska M.A., Dunlop J., Shimpukade B., Christiansen E., Milligan G. (2016). Non-acidic free fatty acid receptor 4 agonists with antidiabetic activity. J. Med. Chem..

[16] Hudson B.D., Shimpukade B., Milligan G., Ulven T. (2014). The molecular basis of ligand interaction at free fatty acid receptor 4 (FFA4/GPR120). J. Biol. Chem..

[17] Liu Z., Hopkins M.M., Zhang Z., Quisenberry C.B., Fix L.C., Galvan B.M., Meier K.E. (2015). Omega-3 fatty acids and other FFA4 agonists inhibit growth factor signaling in human prostate cancer cells. J. Pharmacol. Exp. Ther..

[18] Christiansen E., Due-hansen M.E., Urban C., Grundmann M., Der R.S., Hudson B.D., Milligan G., Cawthorne M.A., Kostenis E., Kassack M.U. (2012). Free fatty acid receptor 1 (FFA1/GPR40) agonists: Mesylpropoxy appendage lowers Lipophilicity and improves ADME properties. J. Med. Chem..

[19] Negoro N., Sasaki S., Ito M., Kitamura S., Tsujihata Y., Ito R., Suzuki M., Takeuchi K., Suzuki N., Miyazaki J. (2012). Identification of fused-ring alkanoic acids with improved pharmacokinetic profiles that act as g protein-coupled receptor 40/ free fatty acid receptor 1 agonists. J. Med. Chem..

[20] Wang X., Zhao T., Yang B., Li Z., Cui J., Dai Y., Qiu Q., Qiang H., Huang W., Qian H. (2015). Synthesis and biological evaluation of phenoxyacetic acid derivatives as novel free fatty acid receptor 1 agonists. Bioorg. Med. Chem..

[21] Wang X., Xu Y., Feng S., Huang X., Meng X., Chen J., Guo L., Ge J., Zhang J., Chen J. (2019). A potent free fatty acid receptor 1 agonist with a glucose-dependent antihyperglycemic effect. Chem. Commun..

[22] Surwit R.S., Kuhn C.M., Cochrane C., McCubbin J.A., Feinglos M.N. (1988). Diet-induced type II diabetes in C57BL/6J mice. Diabetes.

[23] Winzell M.S., Ahrén B. (2004). The high-fat diet–fed mouse: A model for studying mechanisms and treatment of impaired glucose tolerance and type 2 diabetes. Diabetes.

[24] Lee Y., Warne T., Nehmé R., Pandey S., Dwivedi-Agnihotri H., Chaturvedi M., Edwards P.C., García-Nafría J., Leslie A.G., Shukla A.K. (2020). Molecular basis of β-arrestin coupling to formoterol-bound β 1-adrenoceptor. Nature.

[25] Krumm B.E., White J.F., Shah P., Grisshammer R. (2015). Structural prerequisites for G-protein activation by the neurotensin receptor. Nat. Commun..

[26] Rasmussen S.G., Choi H.-J., Fung J.J., Pardon E., Casarosa P., Chae P.S., DeVree B.T., Rosenbaum D.M., Thian F.S., Kobilka T.S. (2011). Structure of a nanobody-stabilized active state of the β 2 adrenoceptor. Nature.

