# Supplementary material for: Novel GPR120 Agonists with Improved Pharmacokinetic Profiles for the Treatment of Type 2 Diabetes
Source: Molecules. 2021 Nov 16;26(22):6907. doi: 10.3390/molecules26226907 (PMC8624523; doi:10.3390/molecules26226907)
Supplement: Supplementary file 1 [file molecules-26-06907-s001.zip › molecules-1432582-supplementary.pdf]

Novel GPR120 agonists with Improved Pharmacokinetic Profiles for the treatment of type 2 diabetes

Guoxia Ji [a,b], Qinghua Guo [a], Qidi Xue [a], Ruifang Kong [a], Shiben Wang [a], Kang Lei [a], Renmin Liu [a]*, and Xuekun Wang [a]*,

[a] School of Pharmaceutical Sciences, Liaocheng University, 1 Hunan Street, Liaocheng, Shandong 252059, China

[b] School of Chemistry and Chemical Engineering, Liaocheng University, 1 Hunan Street, Liaocheng, Shandong 252059, China

* Corresponding author. E-mail addresses: wangxuekun@lcu.edu.cn (X. Wang); Tel.: +86-0635-823-9087 (X.W.)

^{1}H-NMR, ^{13}C-NMR, and HRMS of target compounds

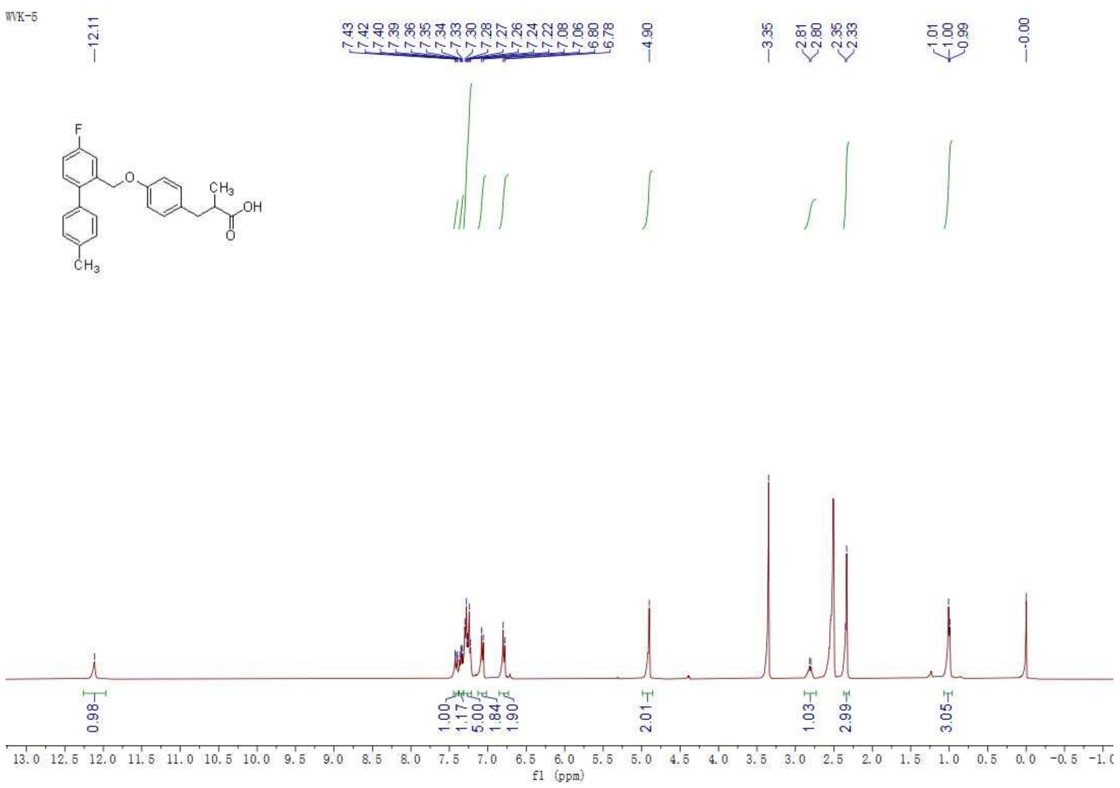

Figure S1. ^{1}H NMR spectrum **1f**.

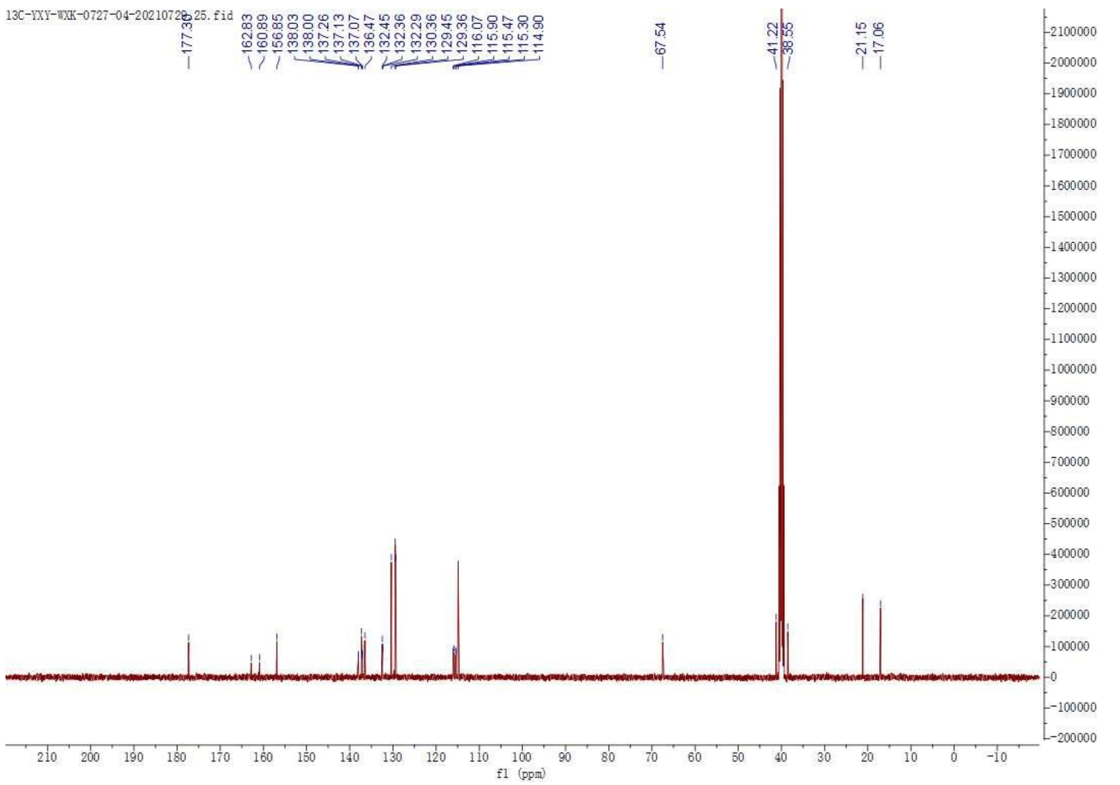

Figure S2. ^{13}C NMR spectrum **1f**.

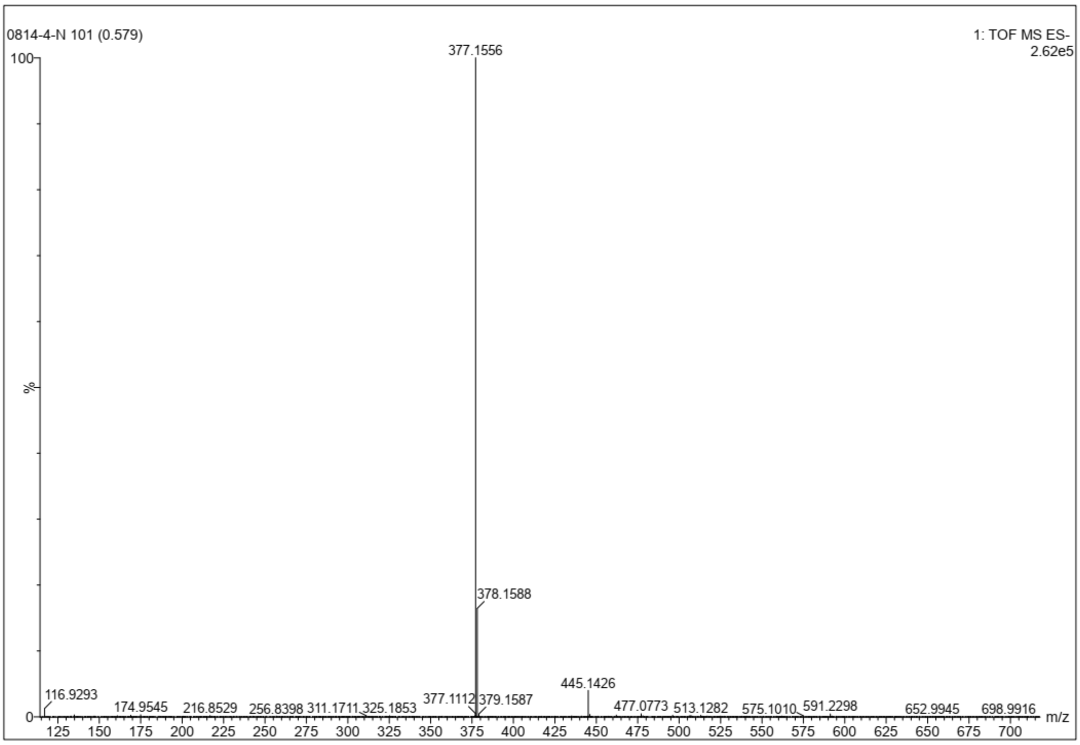

Figure S3. HRMS spectrum **1f**.

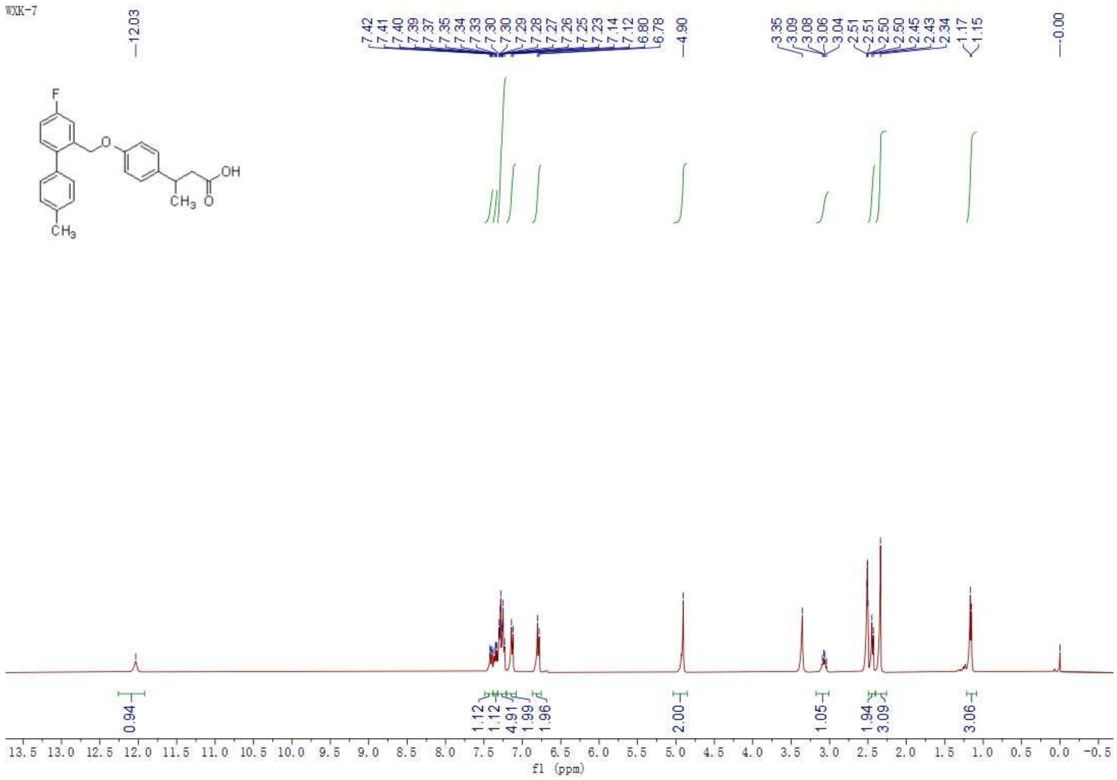

Figure S4. ^{1}H NMR spectrum **2f**.

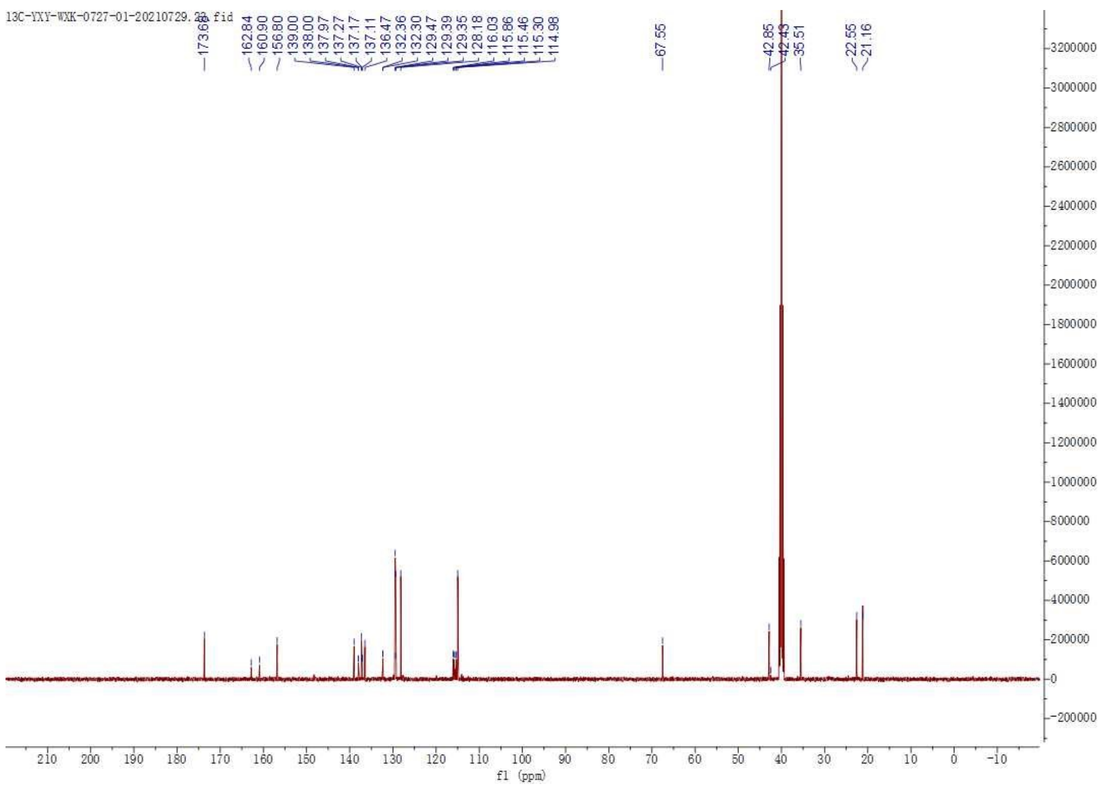

Figure S5. ^{13}C NMR spectrum **2f**.

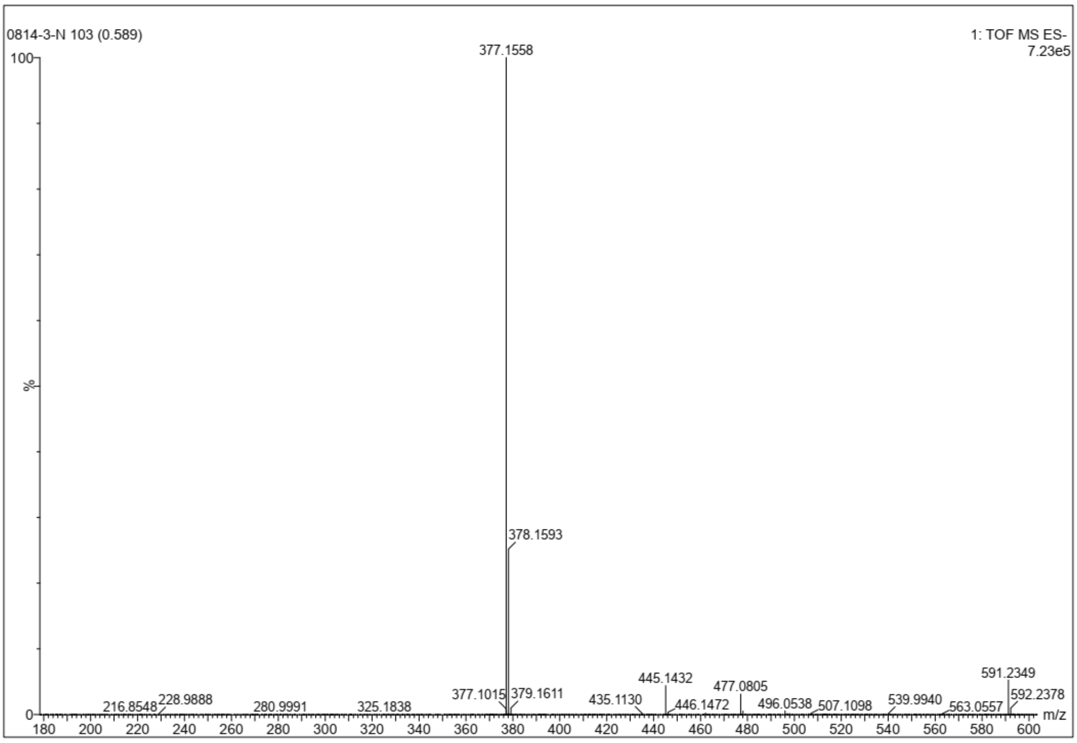

Figure S6. HRMS spectrum **2f**.

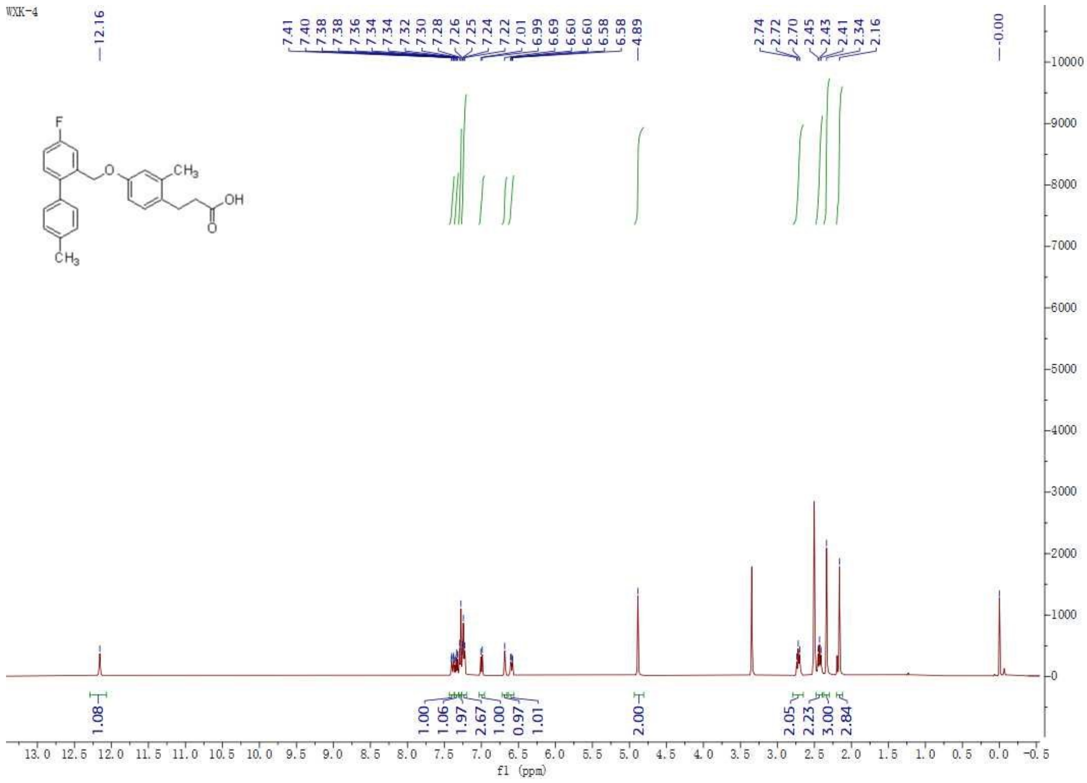

Figure S7. ^{1}H NMR spectrum **3f**.

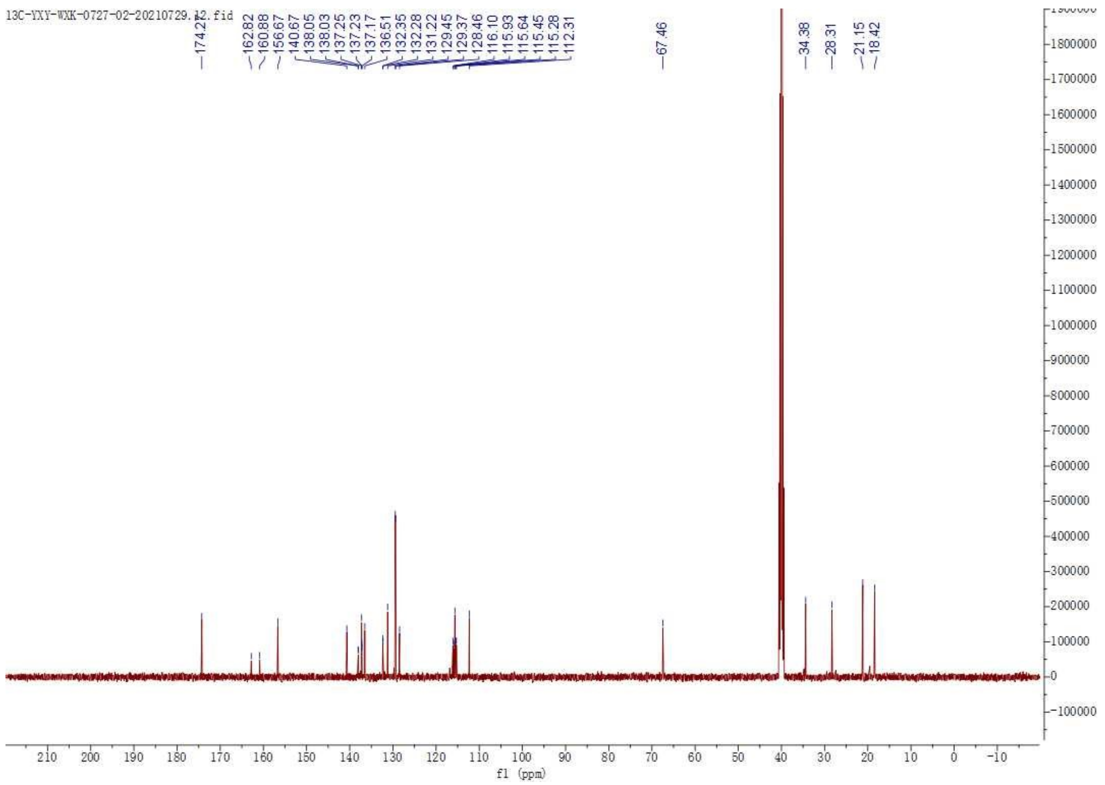

Figure S8. ^{13}C NMR spectrum **3f**.

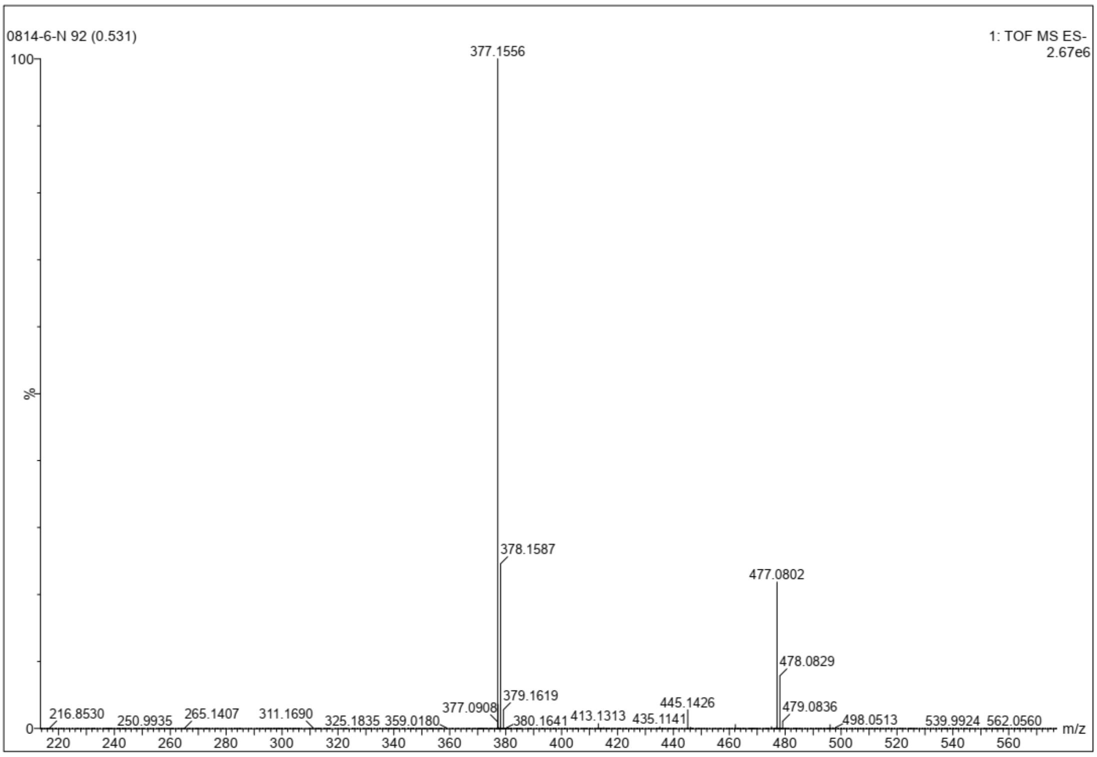

Figure S9. HRMS spectrum **3f**.

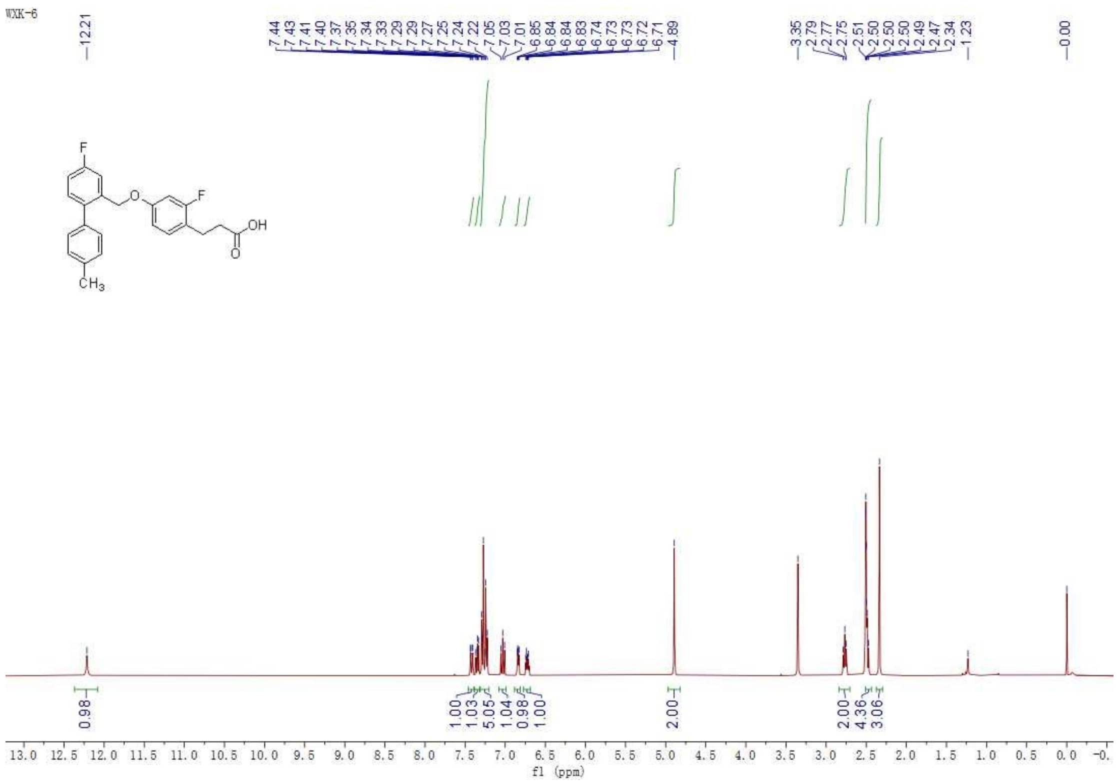

Figure S10. ^{1}H NMR spectrum **4f**.

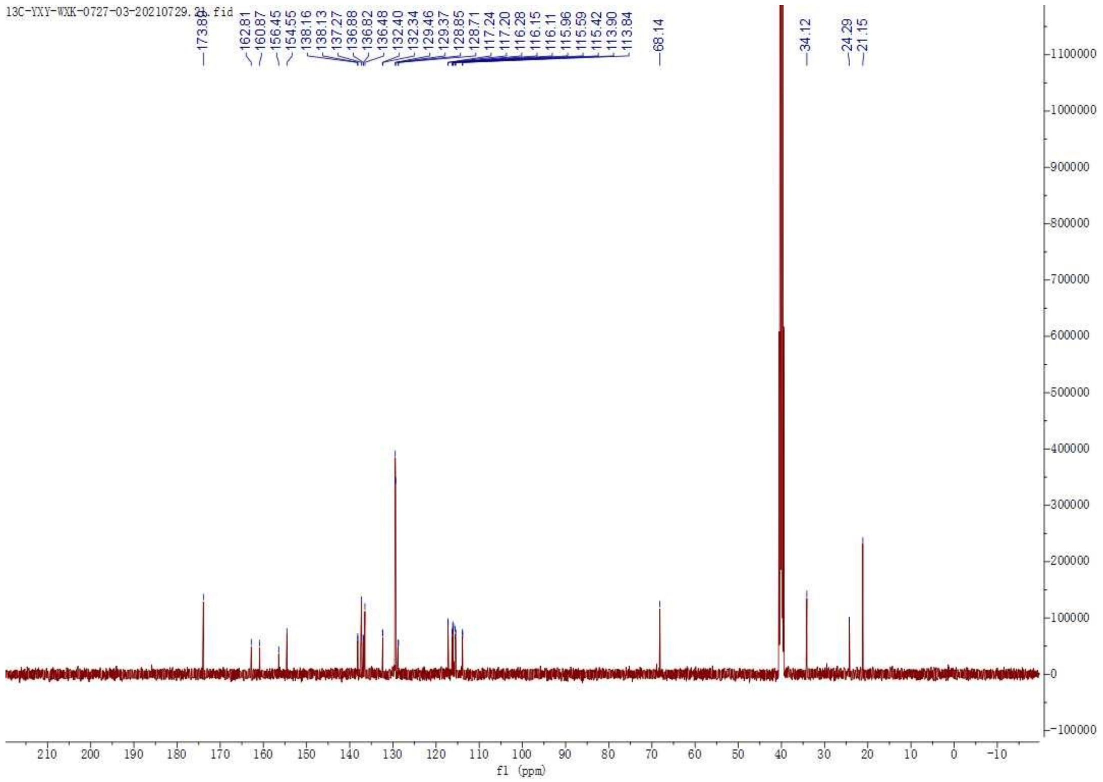

Figure S11. ^{13}C NMR spectrum **4f**.

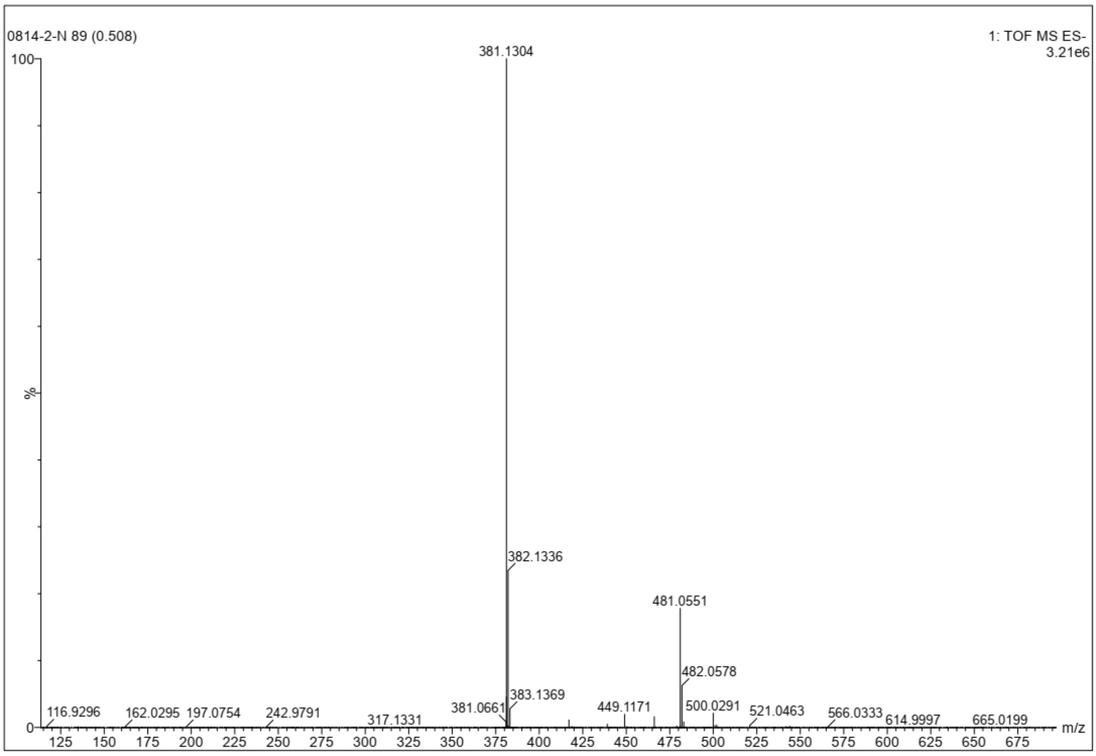

Figure S12. HRMS spectrum **4f**.

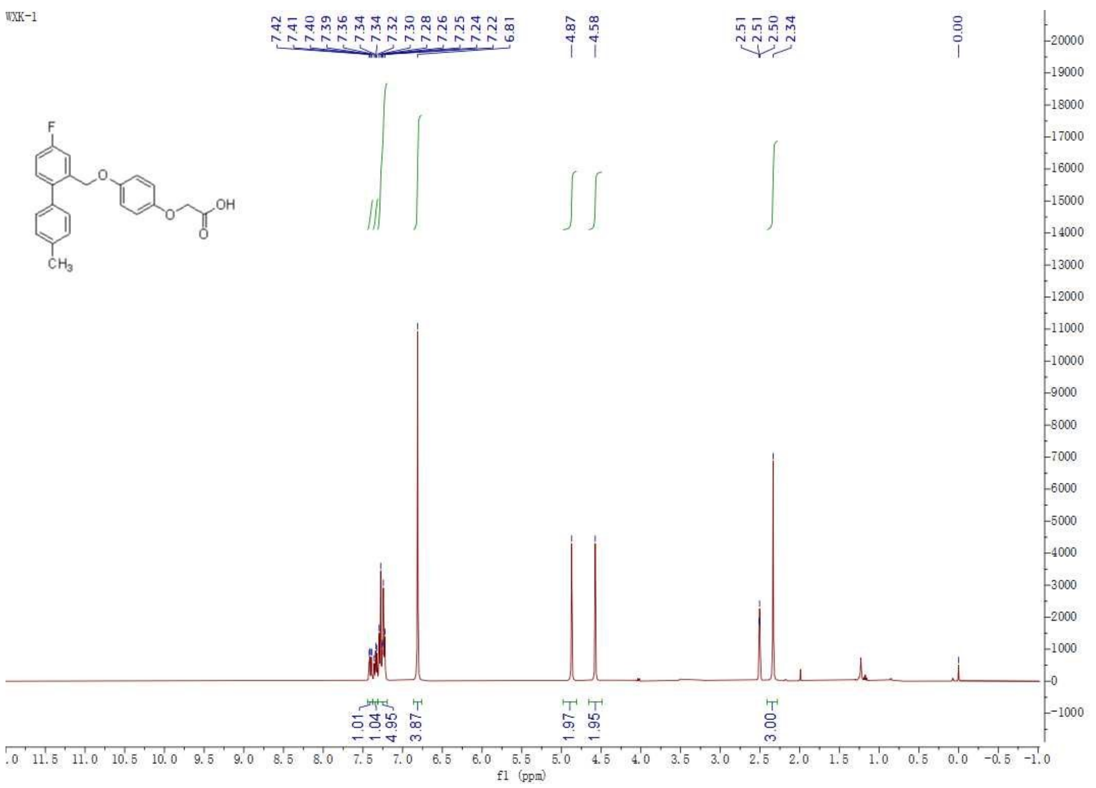

Figure S13. ^{1}H NMR spectrum **5f**.

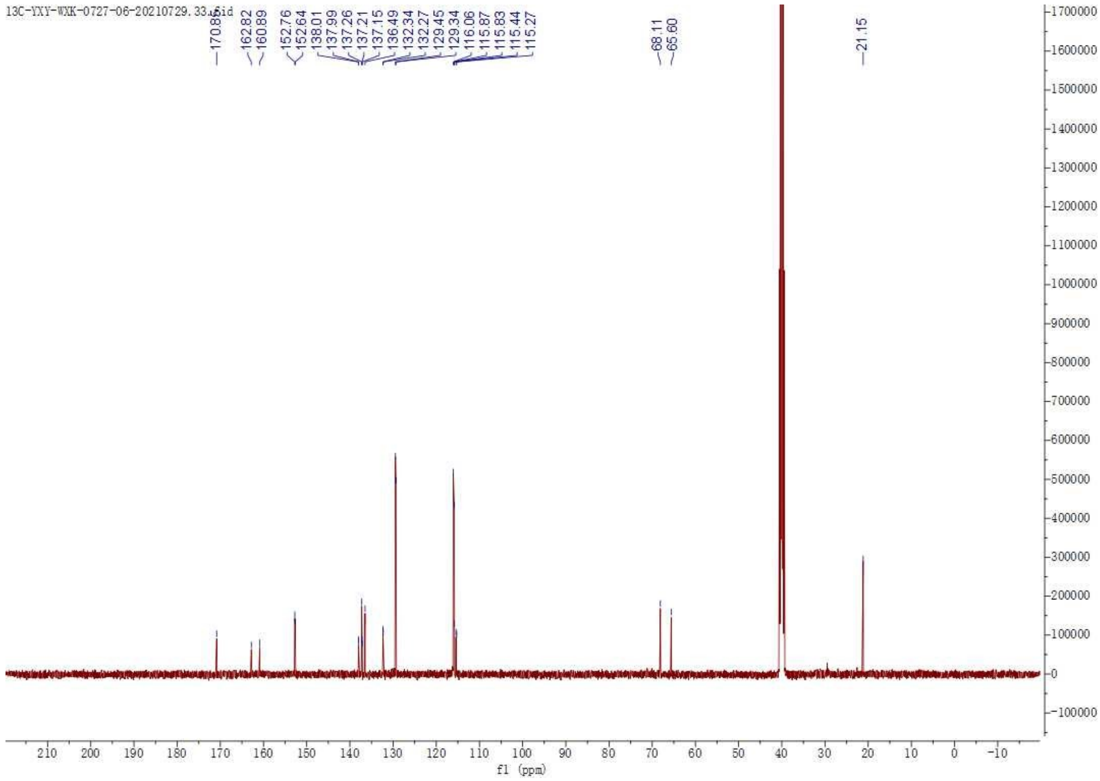

Figure S14. ^{13}C NMR spectrum **5f**.

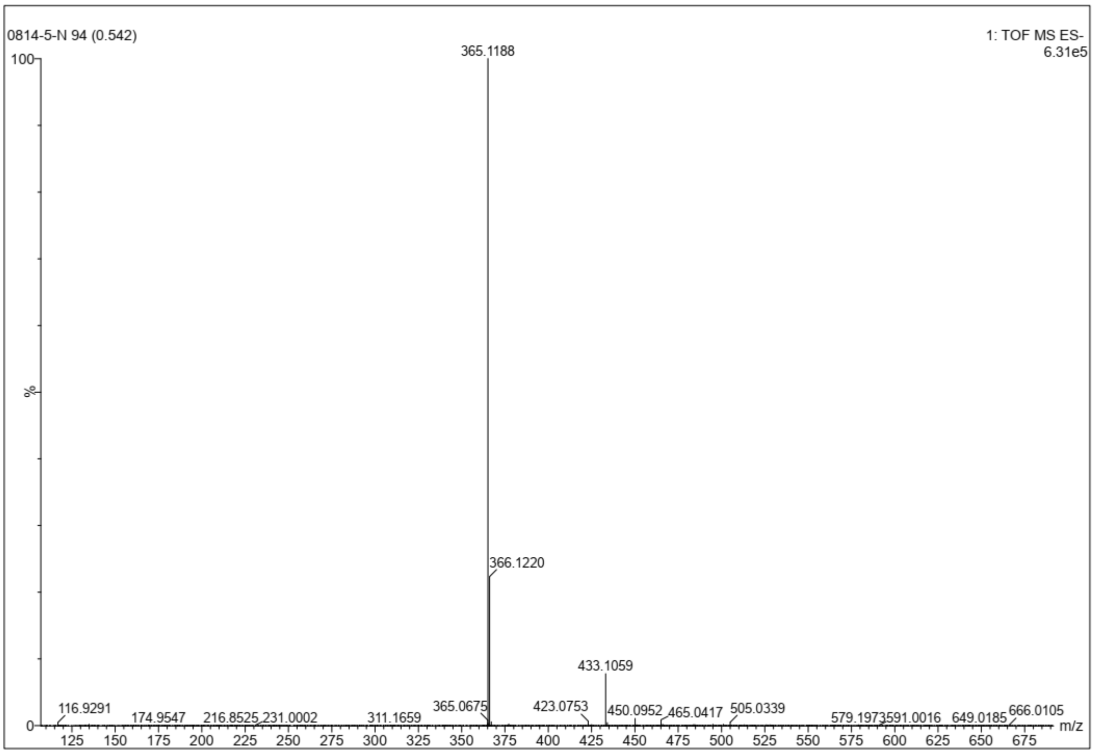

Figure S15. HRMS spectrum **5f**.

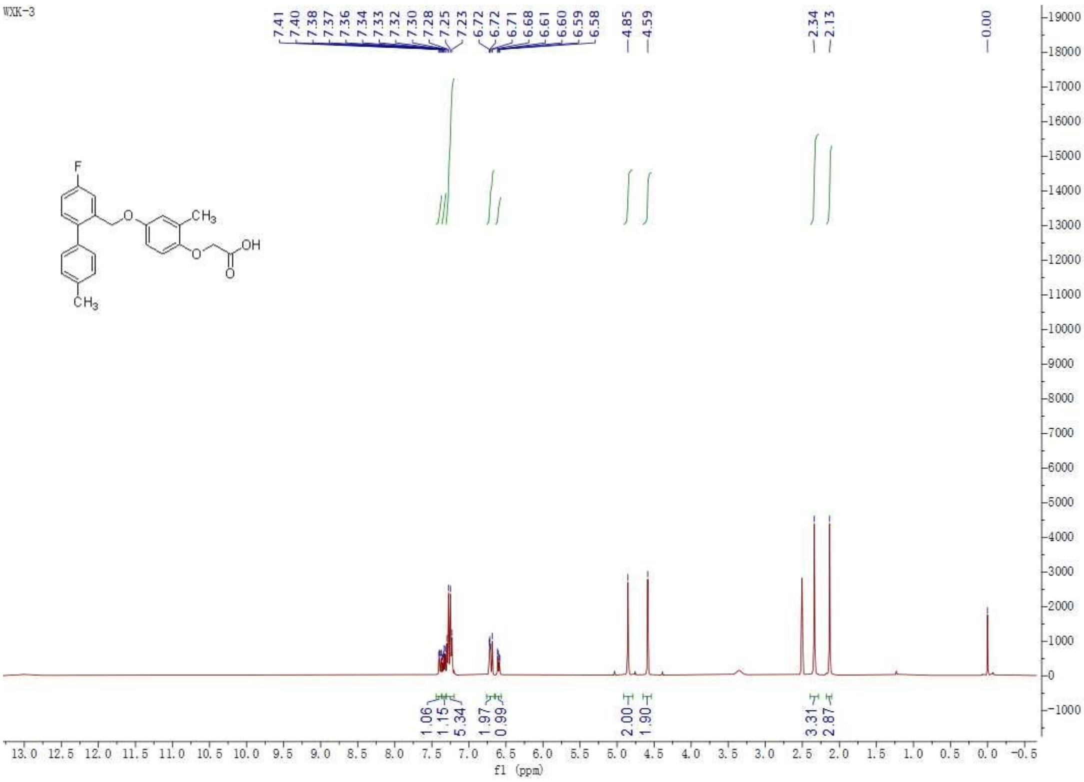

Figure S16. ^{1}H NMR spectrum **6f**.

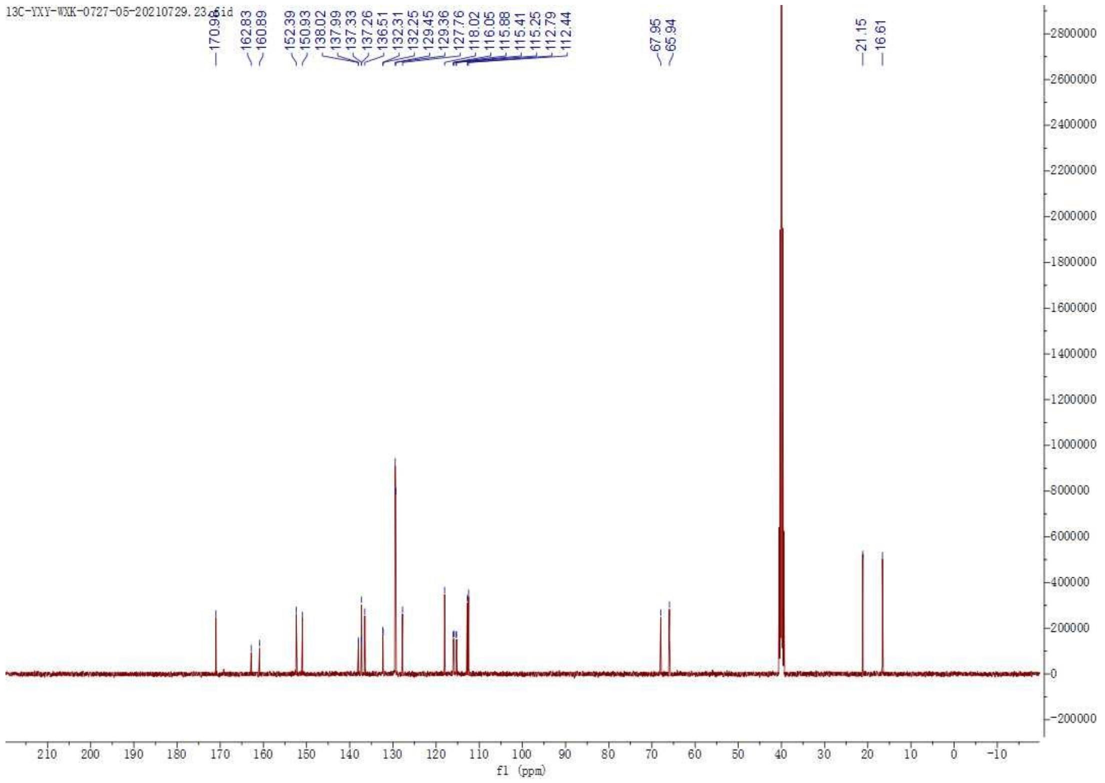

Figure S17. ^{13}C NMR spectrum **6f**.

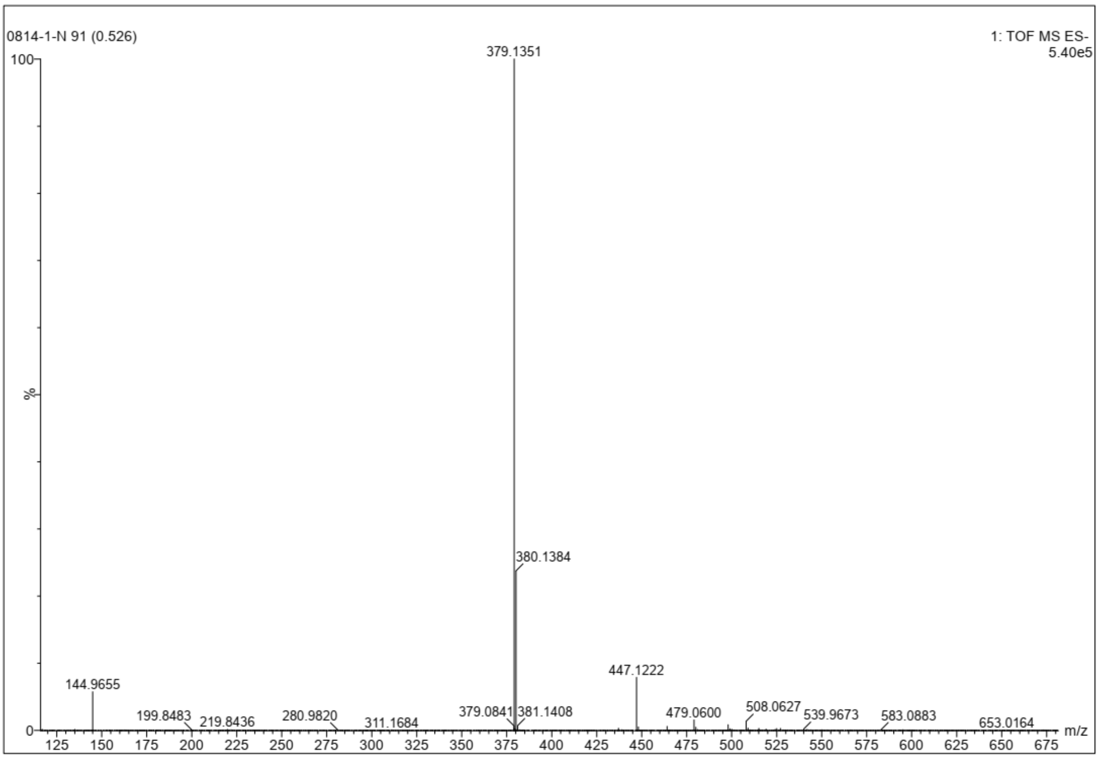

Figure S18. HRMS spectrum **6f**.

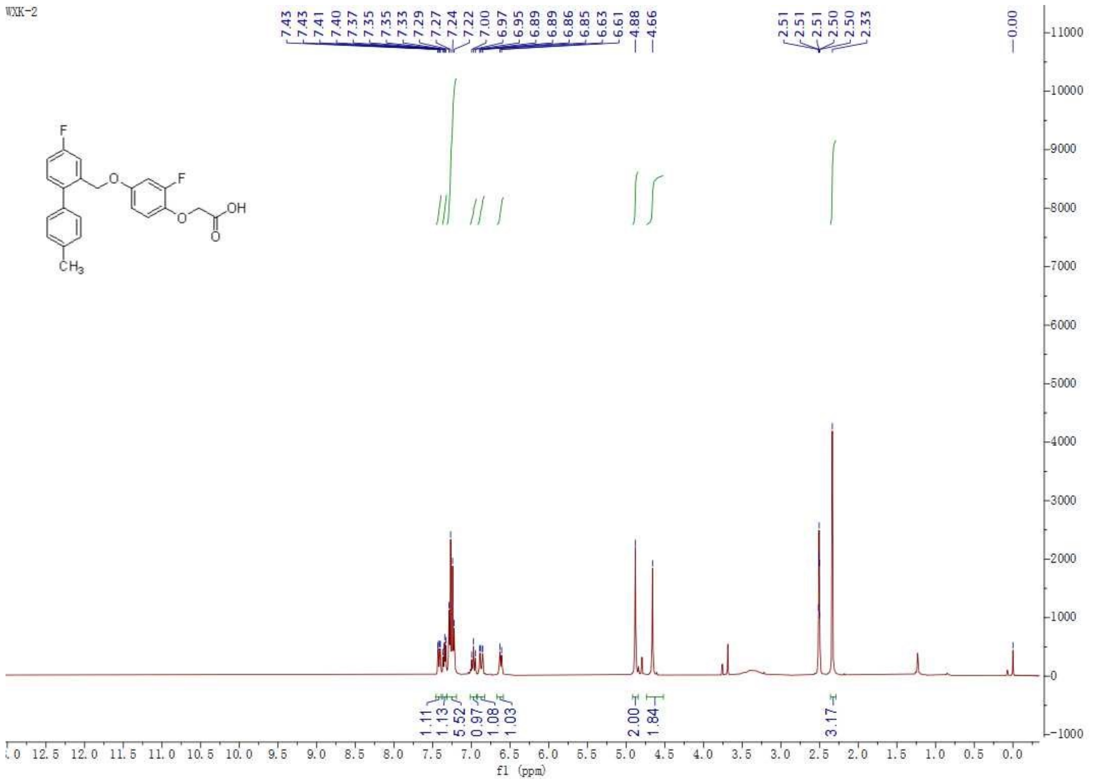

Figure S19. ^{1}H NMR spectrum **7f**.

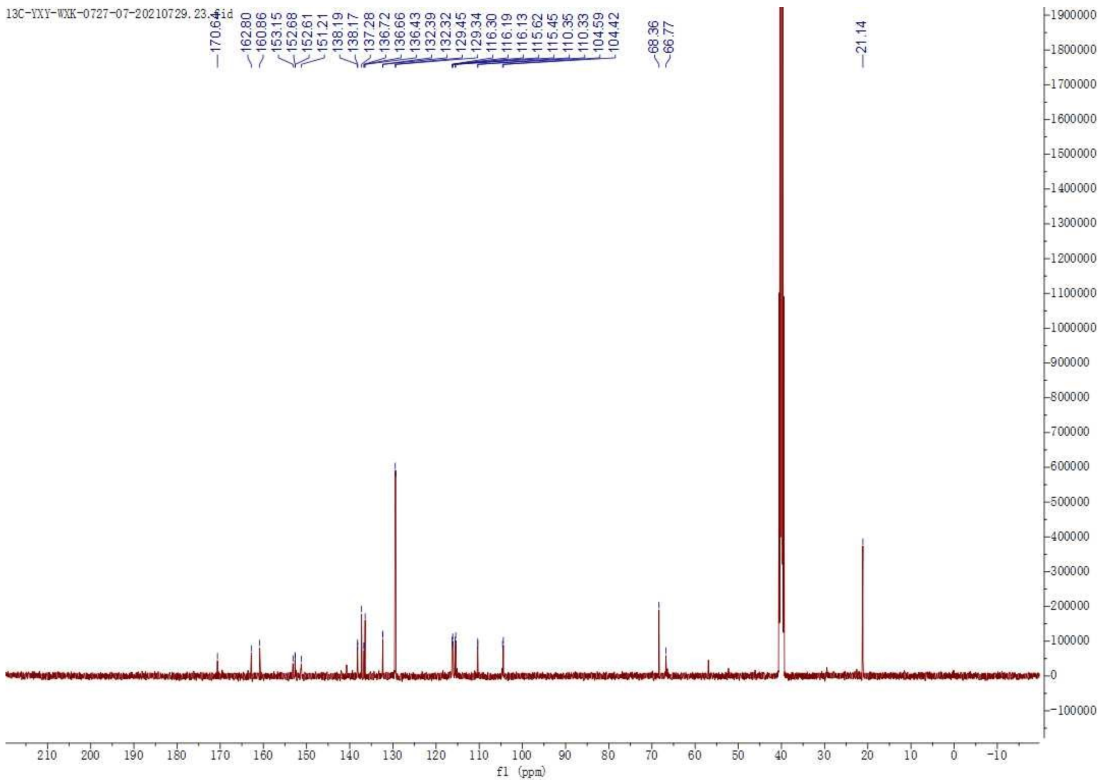

Figure S20. ^{13}C NMR spectrum **7f**.

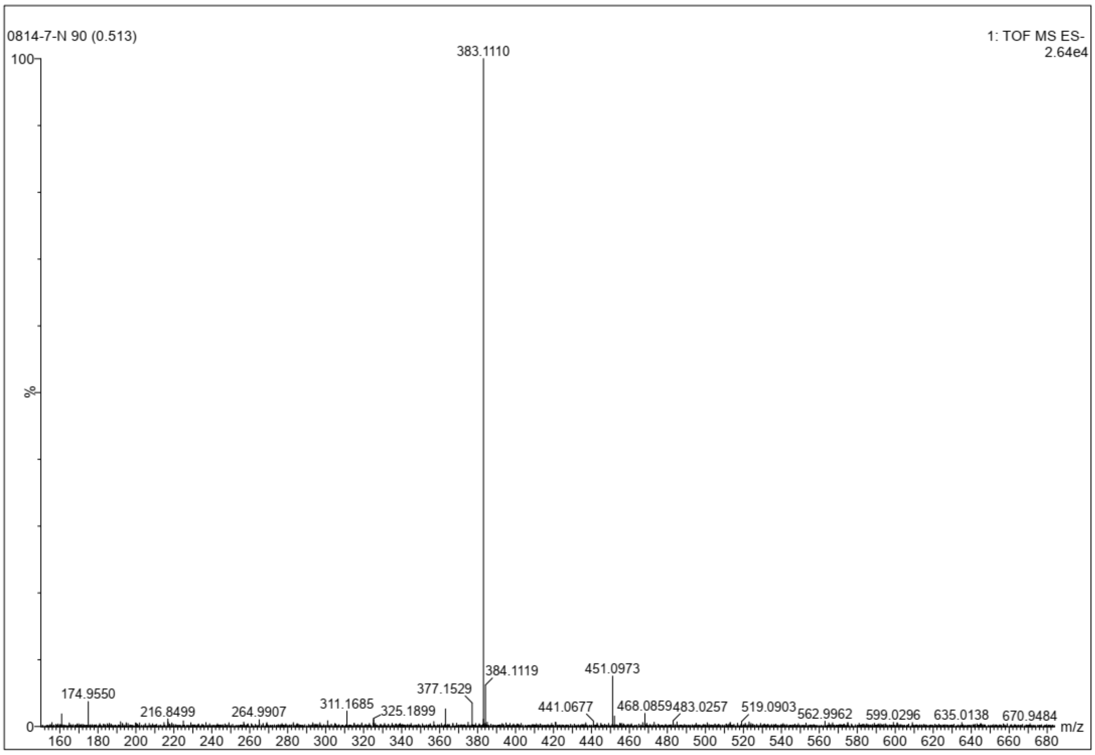

Figure S21. HRMS spectrum **7f**.

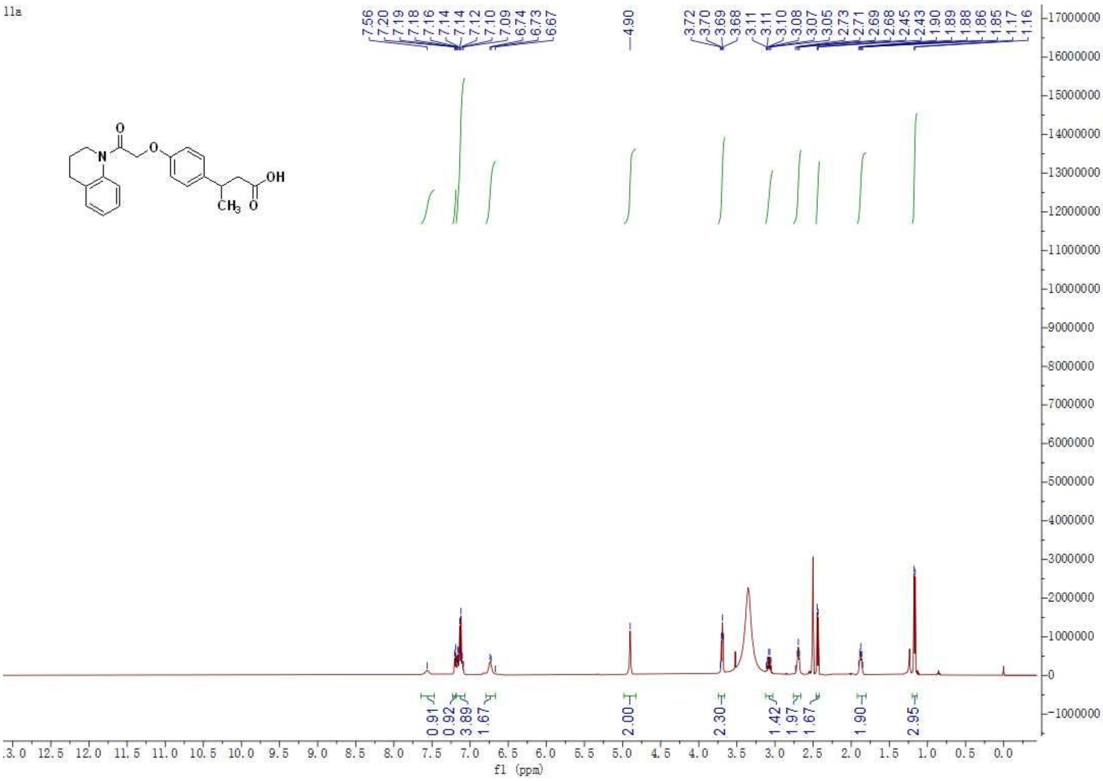

Figure S22. ^{1}H NMR spectrum **11a**.

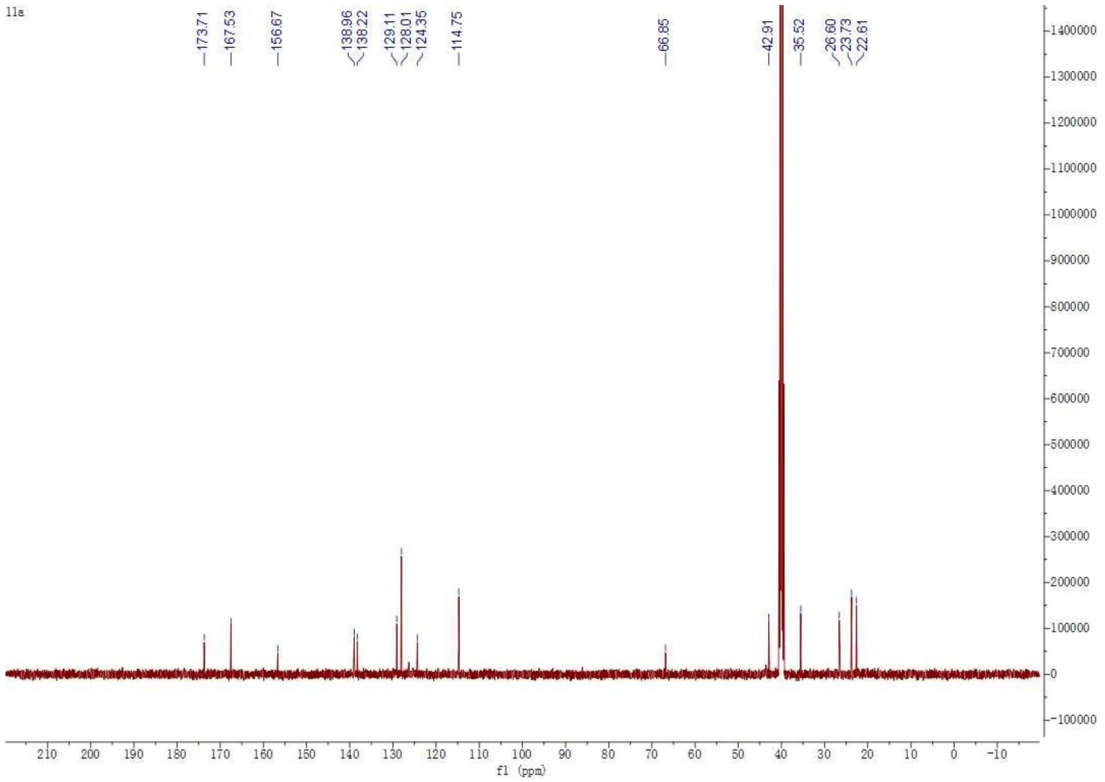

Figure S23. ^{13}C NMR spectrum **11a**.

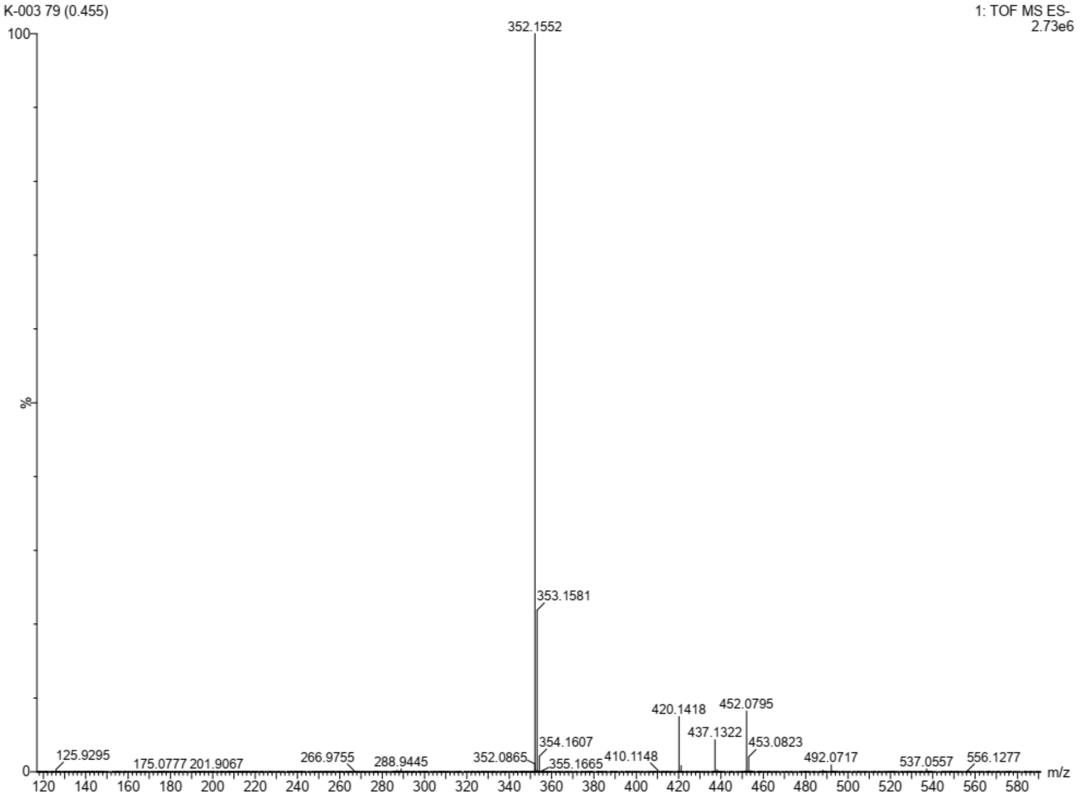

Figure S24. HRMS spectrum **11a**.

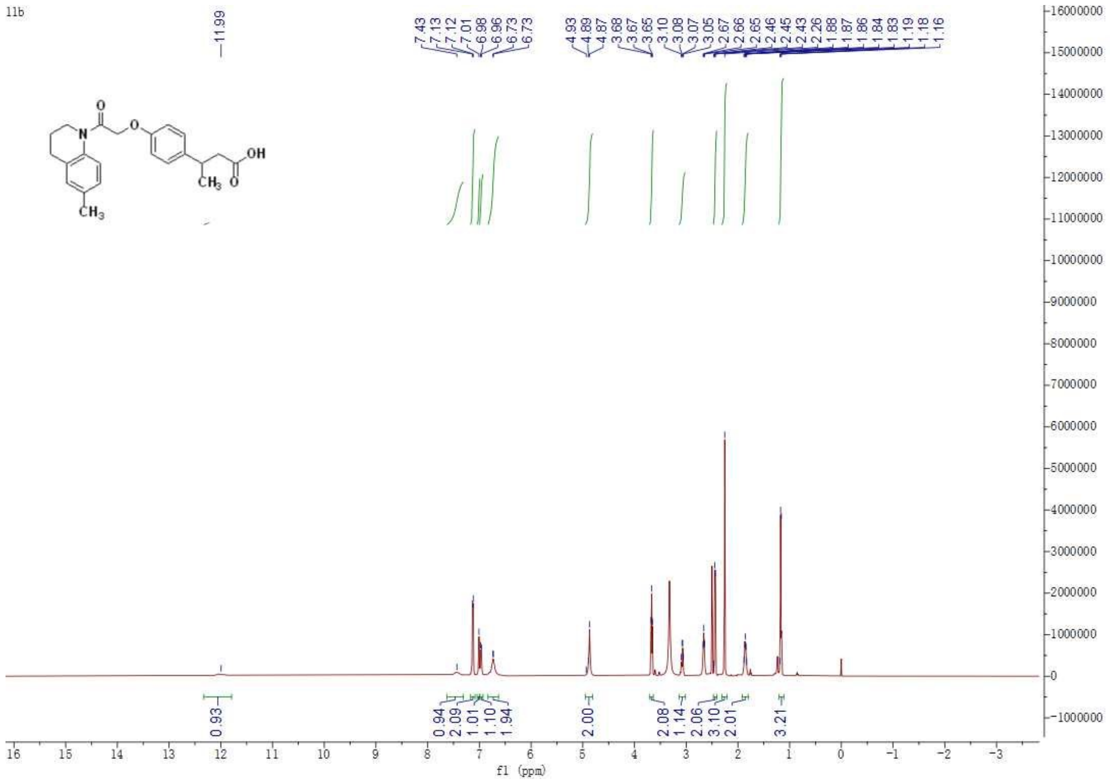

Figure S25. ^{1}H NMR spectrum **11b**.

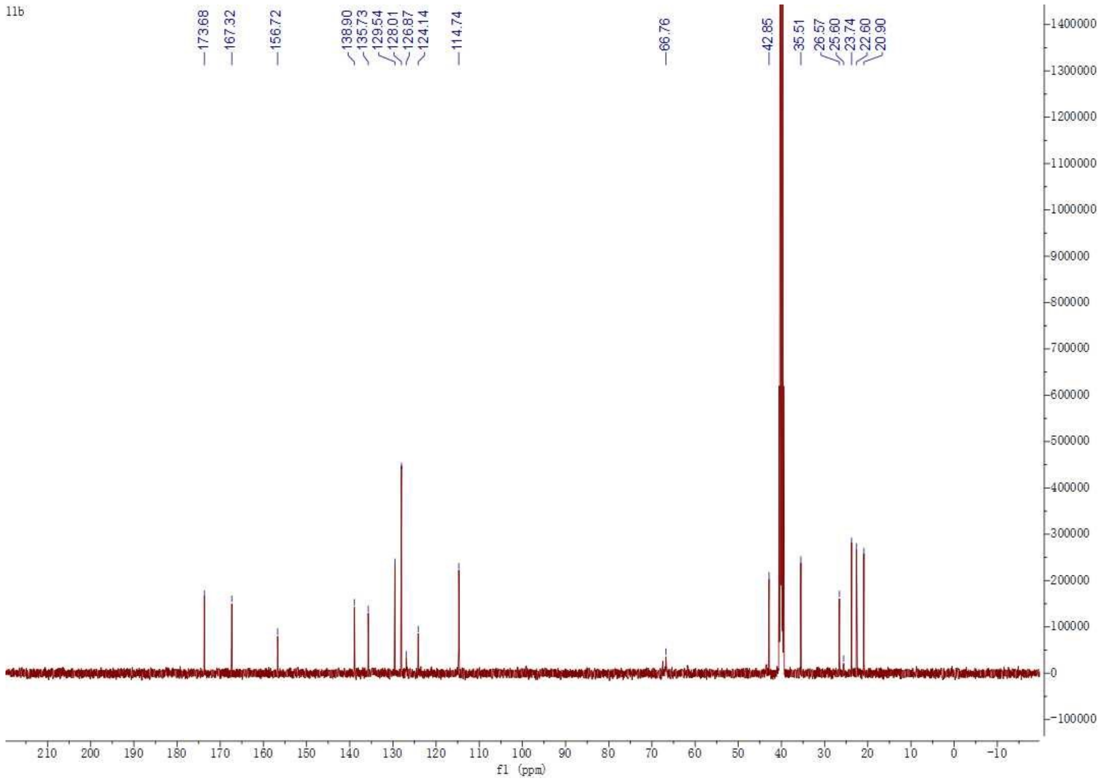

Figure S26. ^{13}C NMR spectrum **11b**.

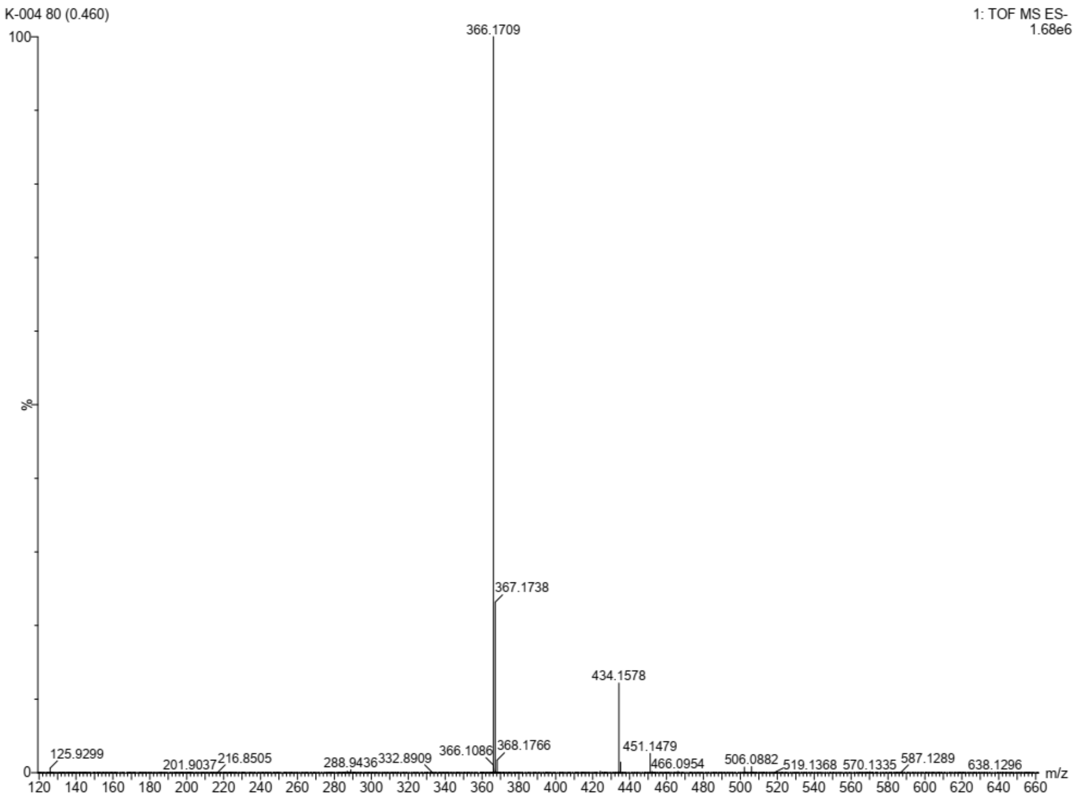

Figure S27. HRMS spectrum **11b**.

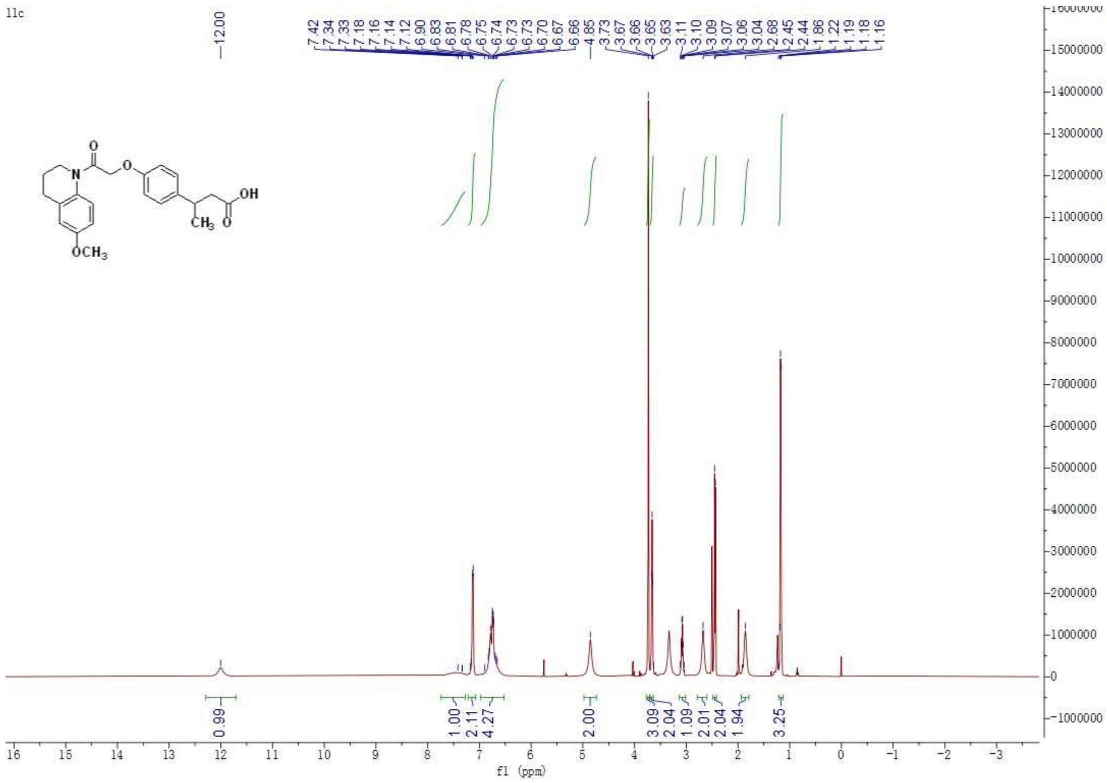

Figure S28. ^{1}H NMR spectrum **11c**.

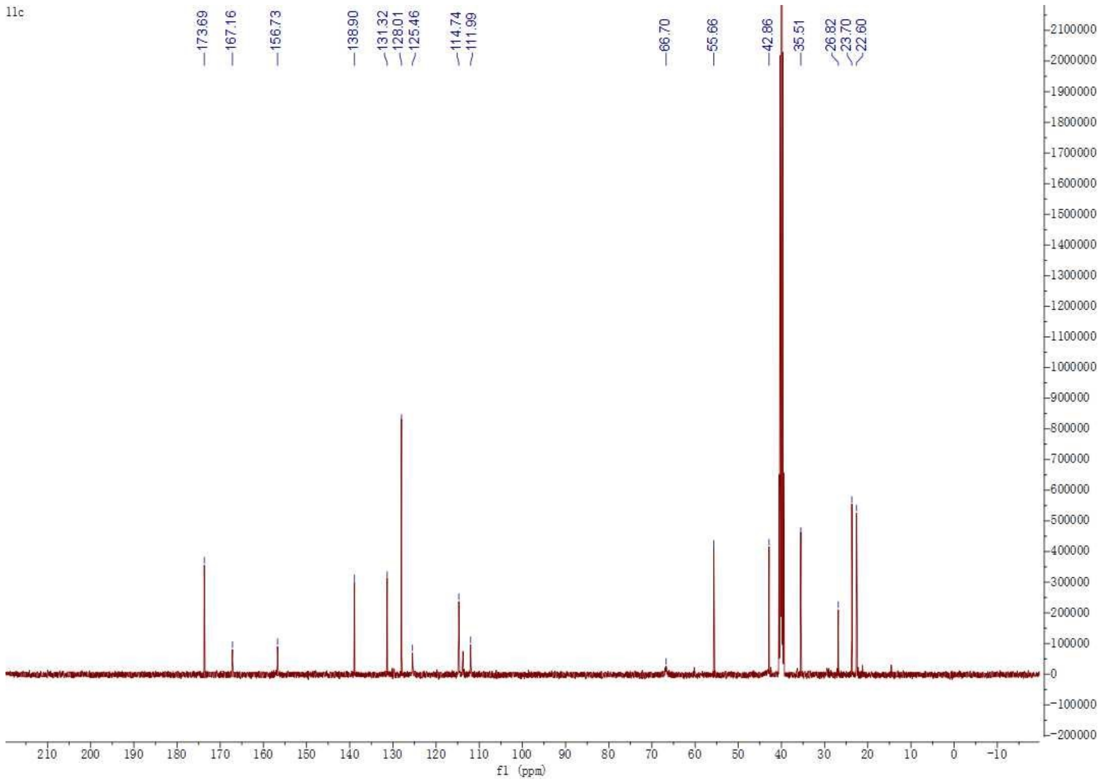

Figure S29. ^{13}C NMR spectrum **11c**.

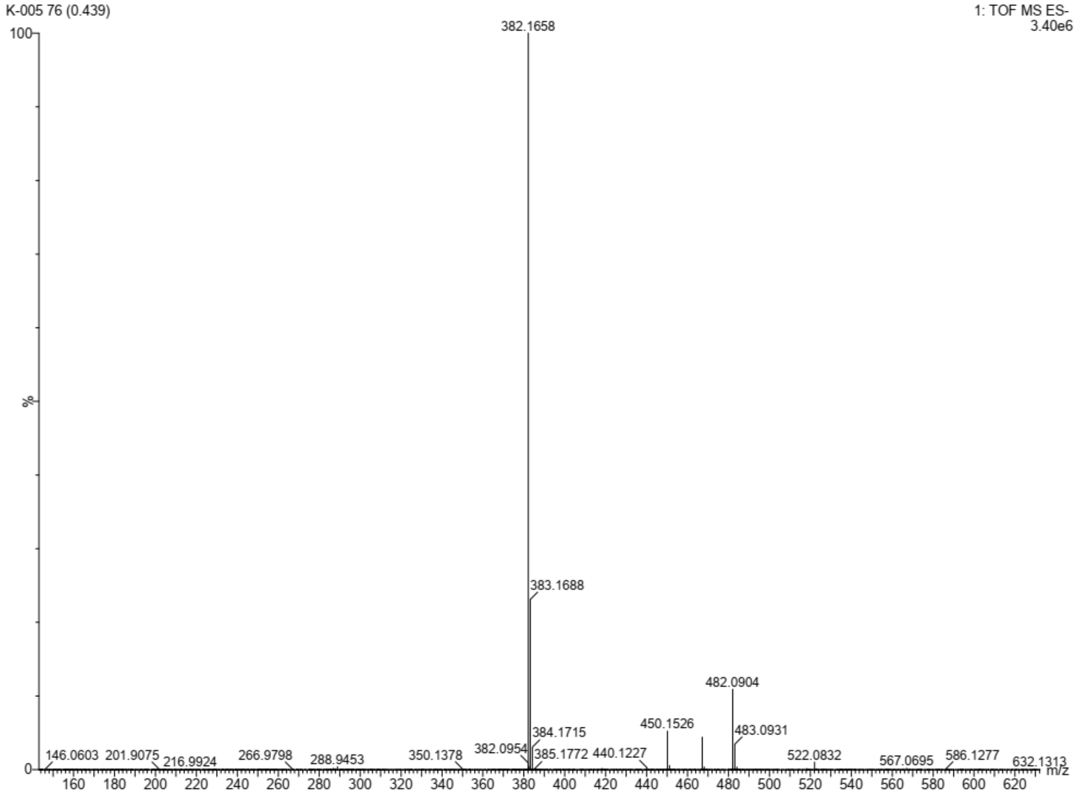

Figure S30. HRMS spectrum **11c**.

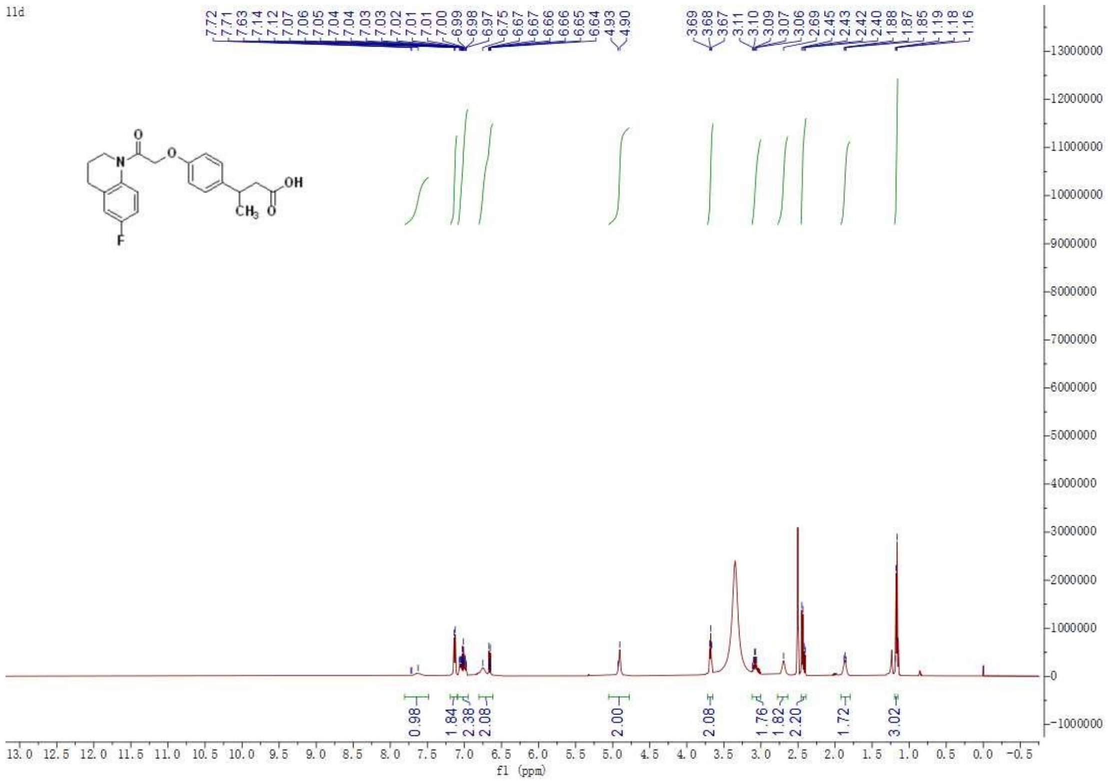

Figure S31. ^{1}H NMR spectrum **11d**.

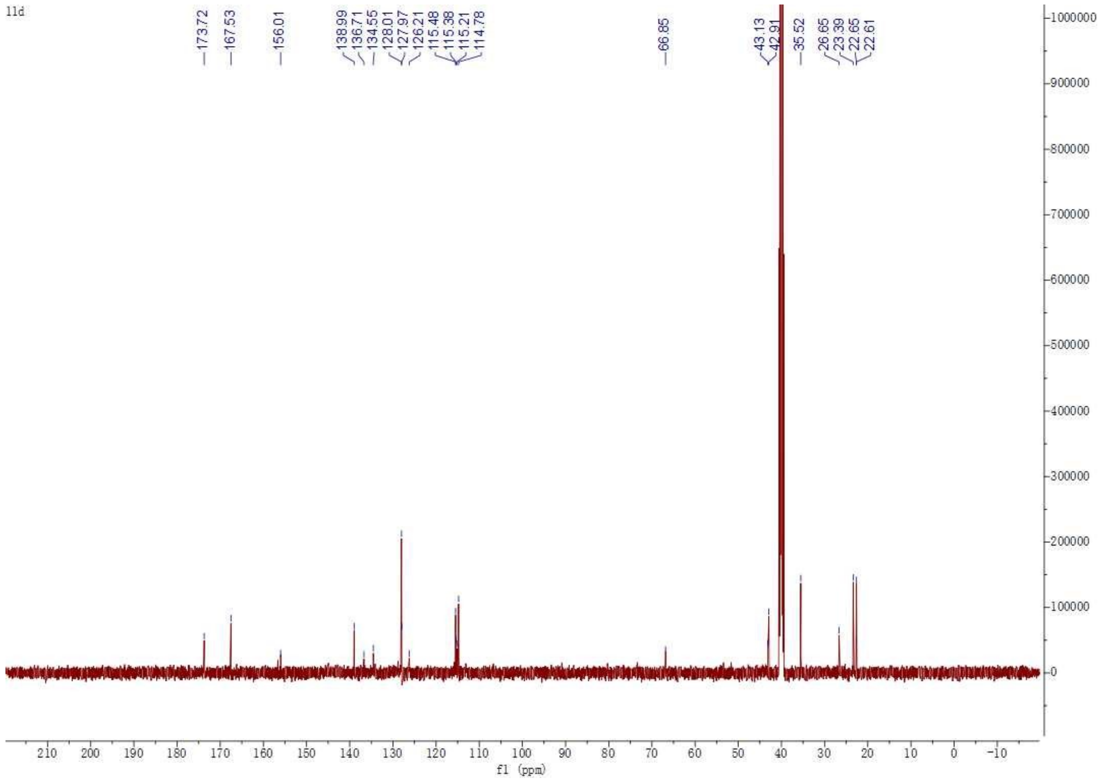

Figure S32. ^{13}C NMR spectrum **11d**.

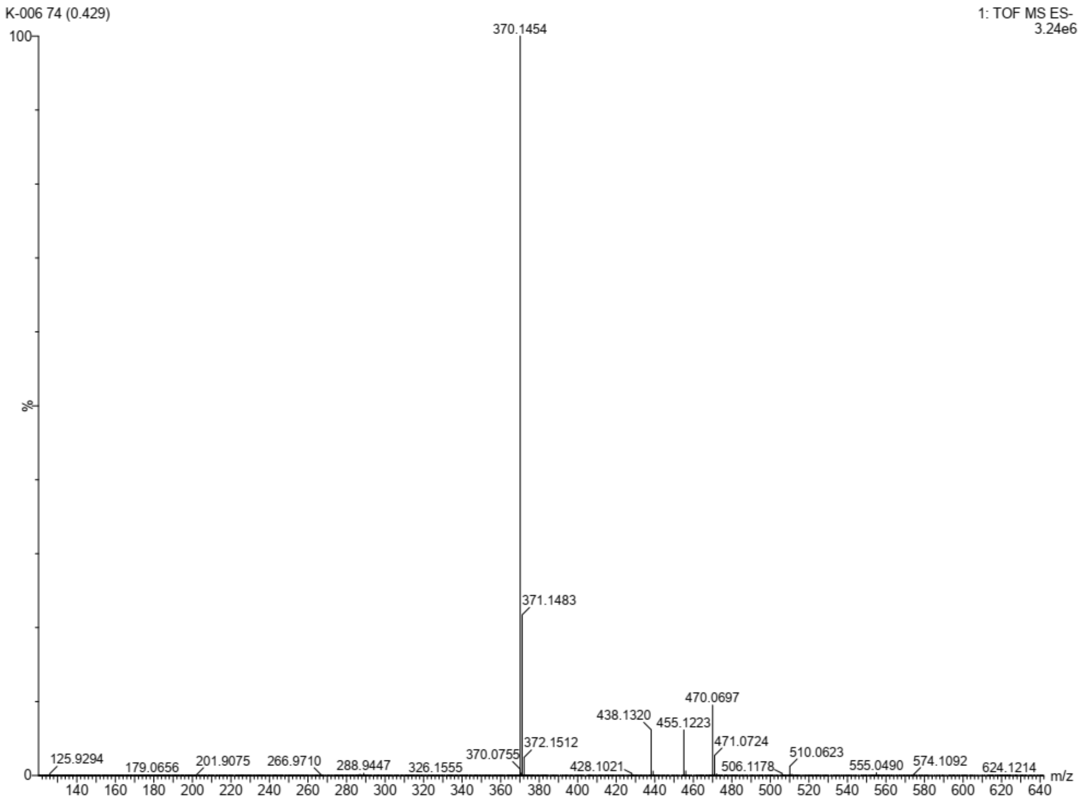

Figure S33. HRMS spectrum **11d**.

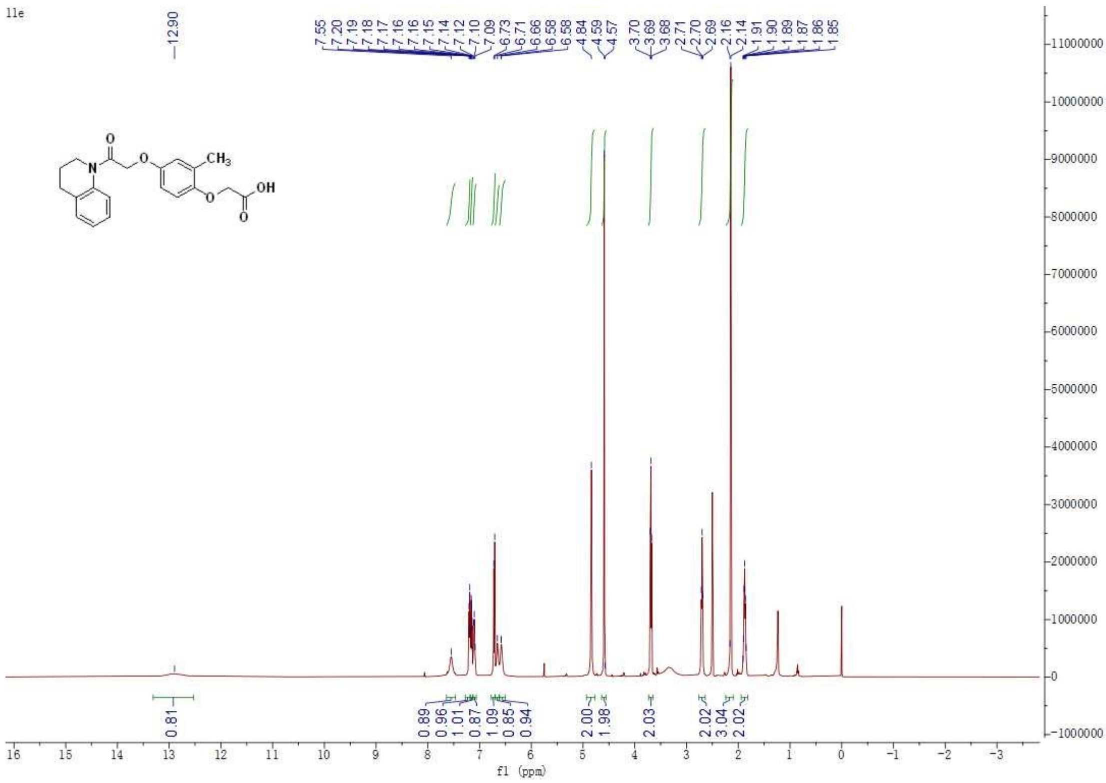

Figure S34. ^{1}H NMR spectrum **11e**.

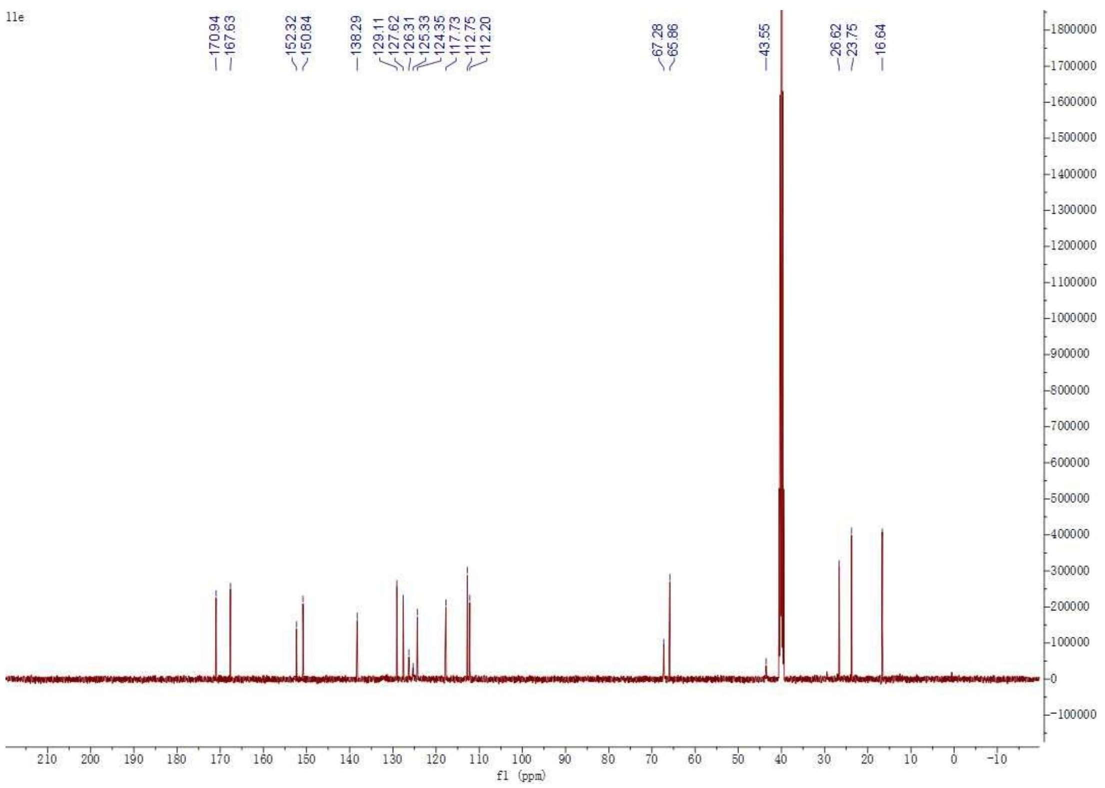

Figure S35. ^{13}C NMR spectrum **11e**.

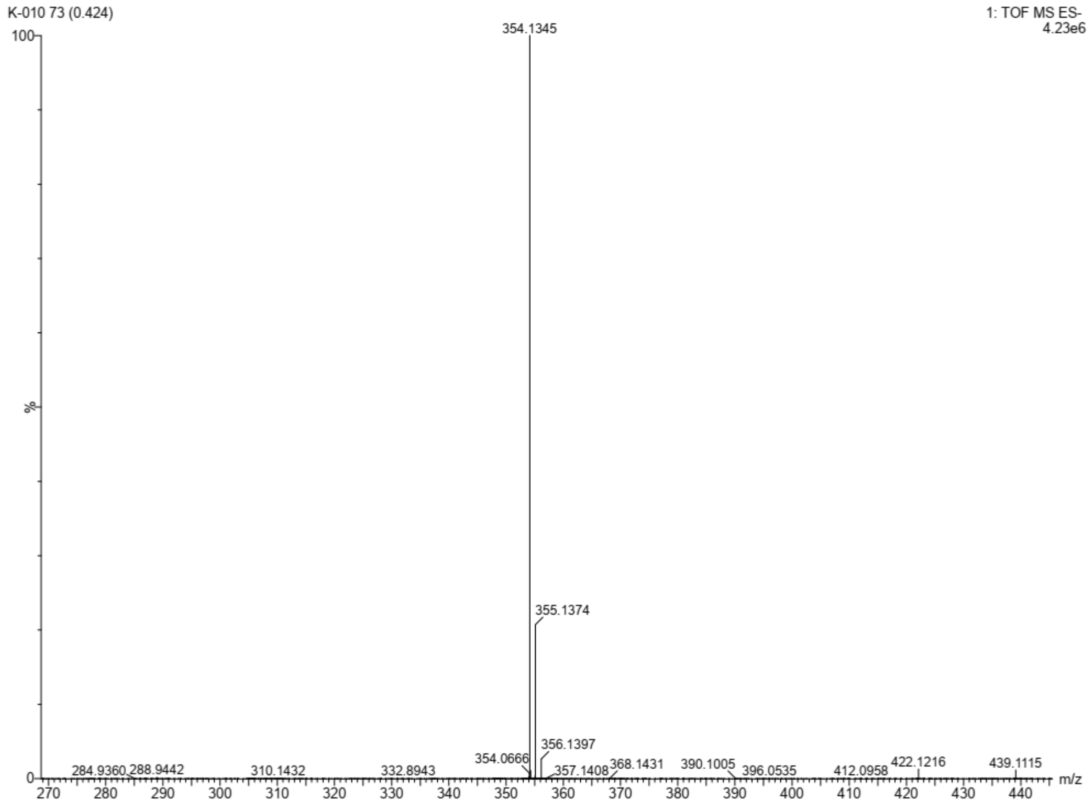

Figure S36. HRMS spectrum **11e**.

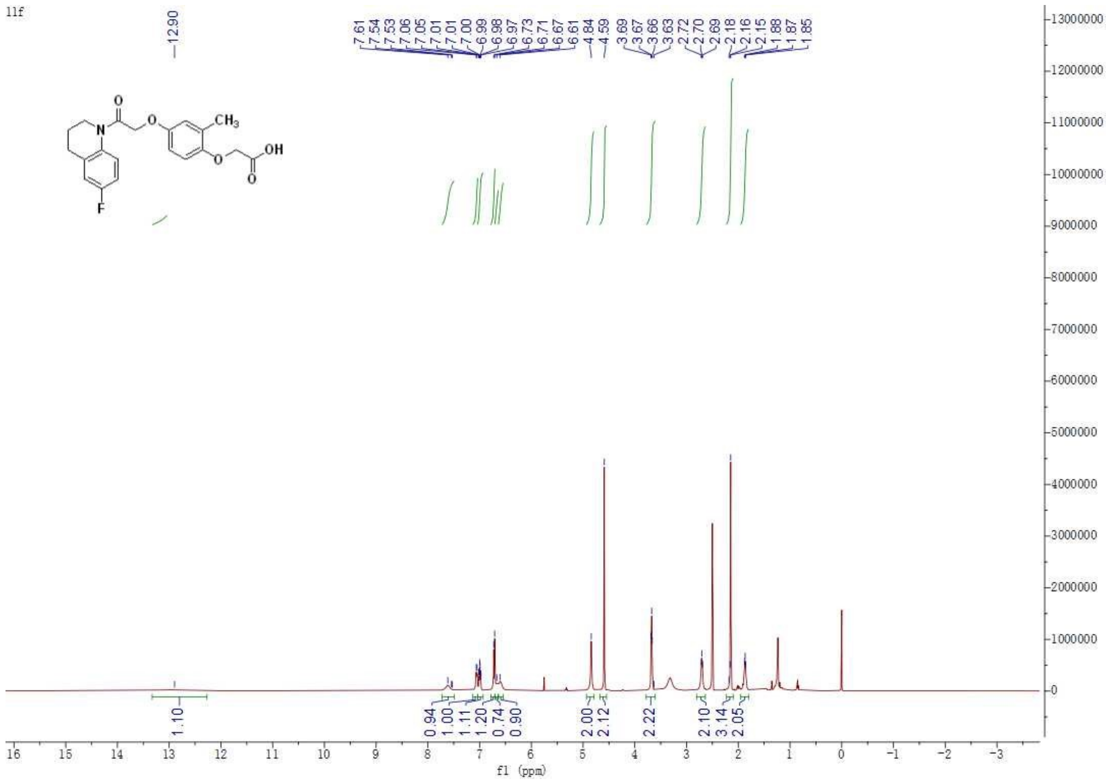

Figure S37. ^{1}H NMR spectrum **11f**.

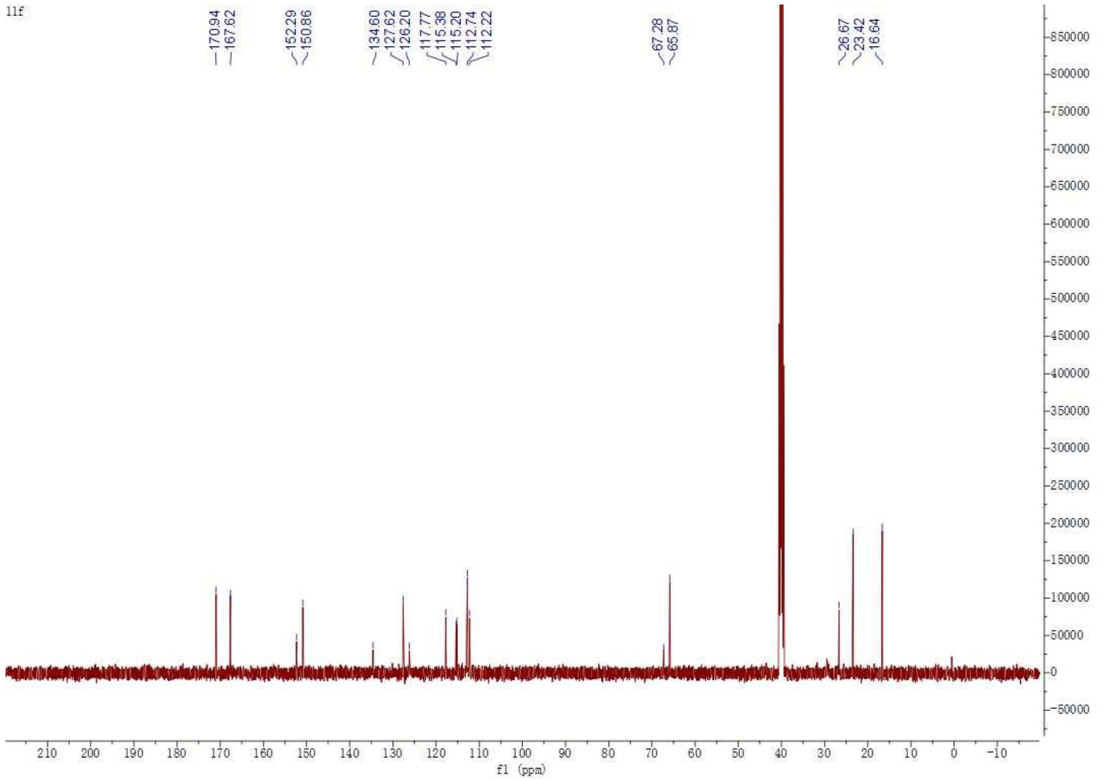

Figure S38. ^{13}C NMR spectrum **11f**.

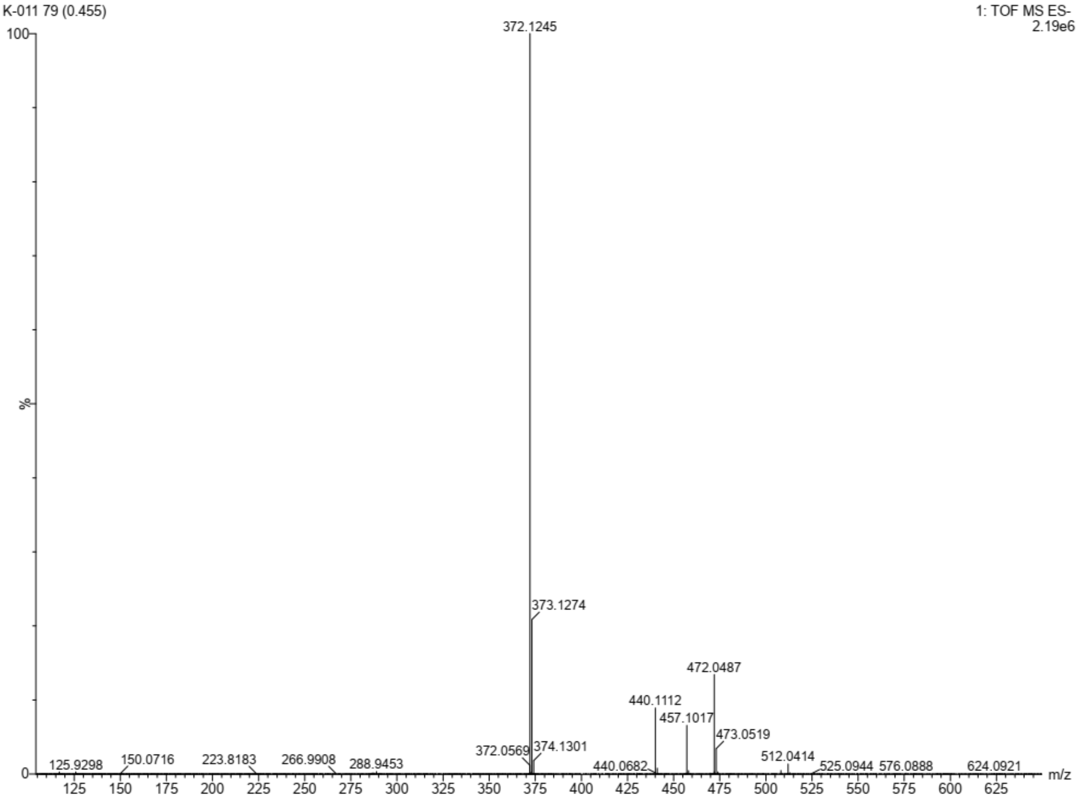

Figure S39. HRMS spectrum **11f**.

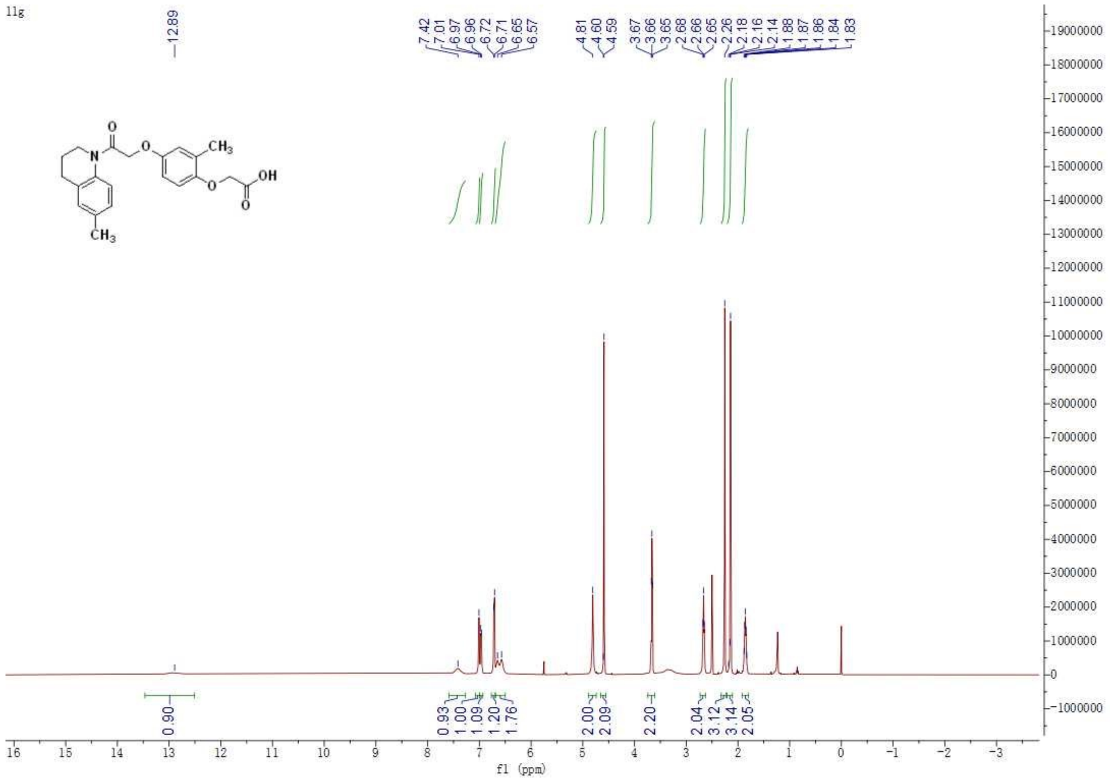

Figure S40. ^{1}H NMR spectrum **11g**.

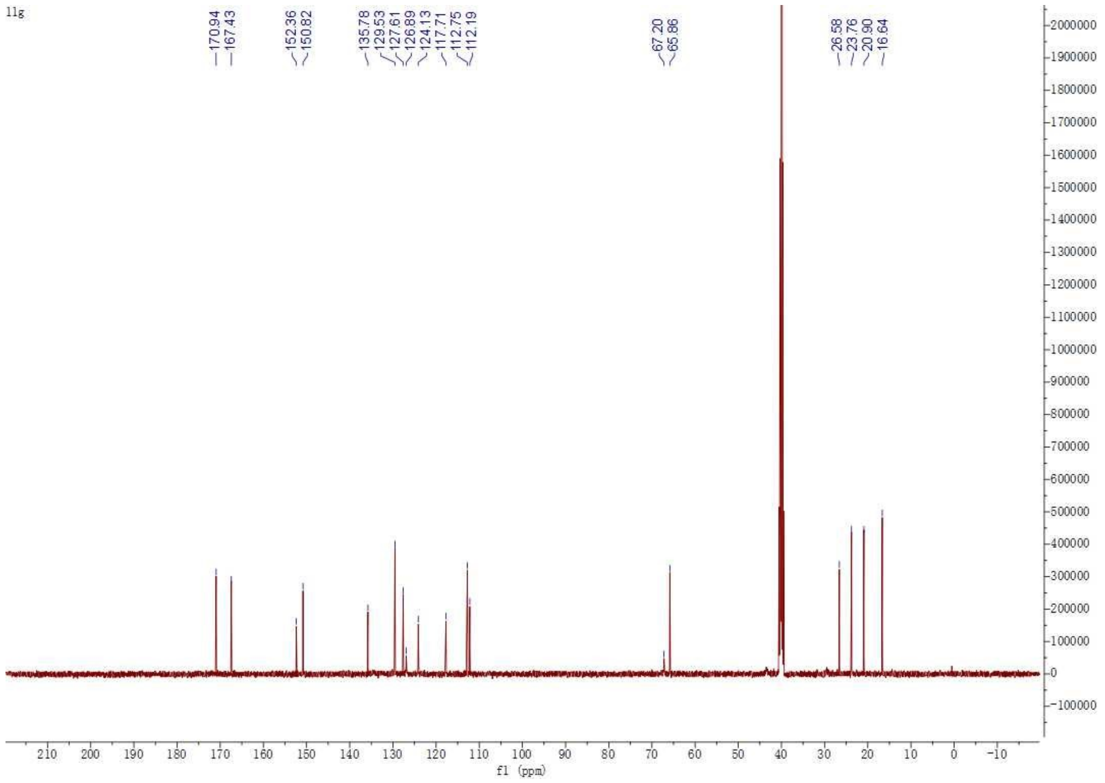

Figure S41. ^{13}C NMR spectrum **11g**.

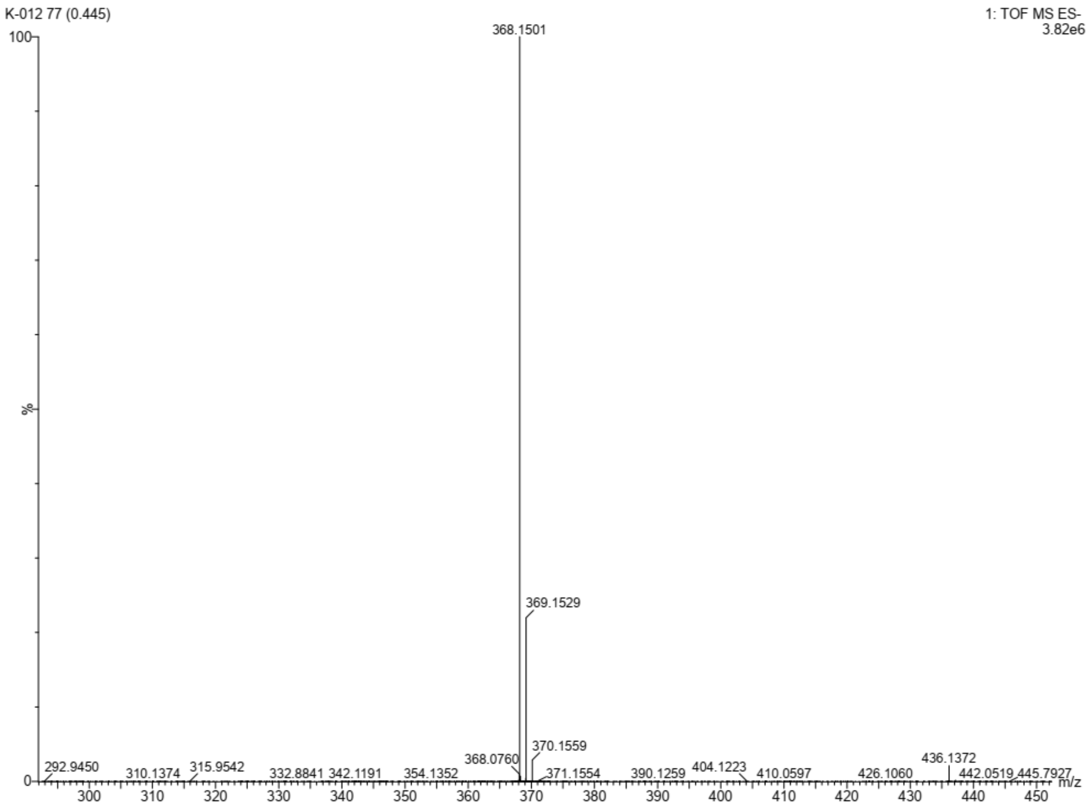

Figure S42. HRMS spectrum **11g**.

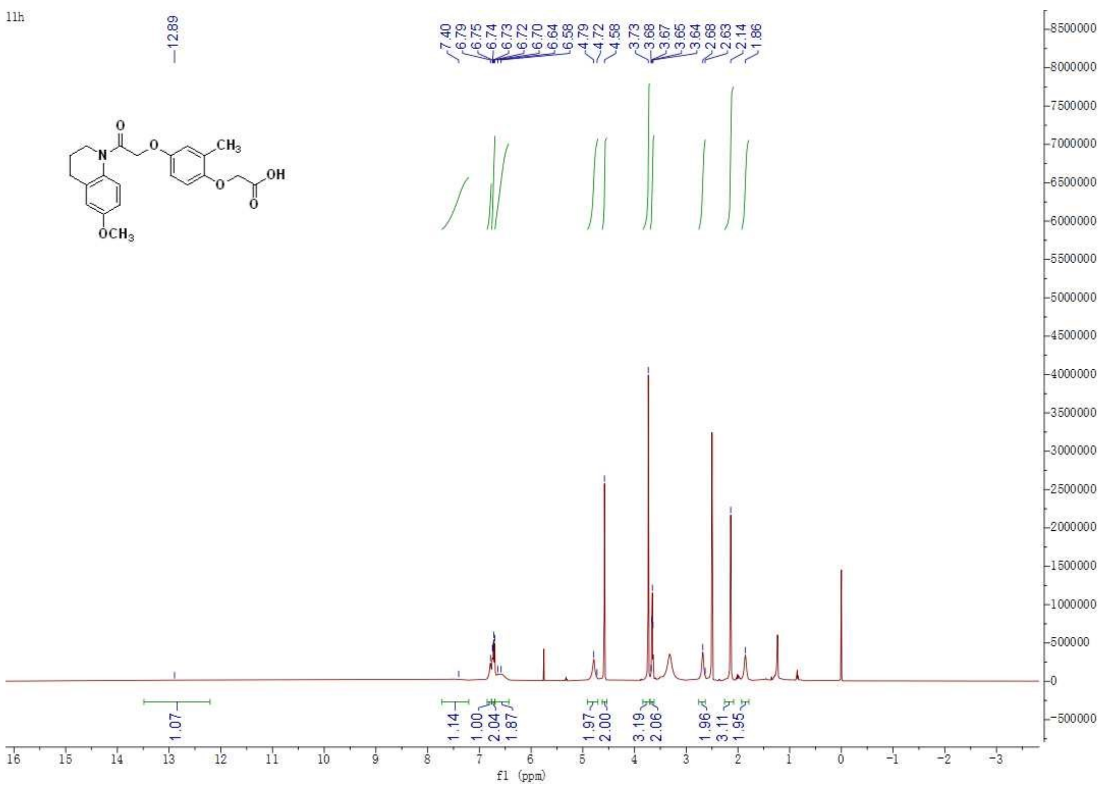

Figure S43. ^{1}H NMR spectrum **11h**.

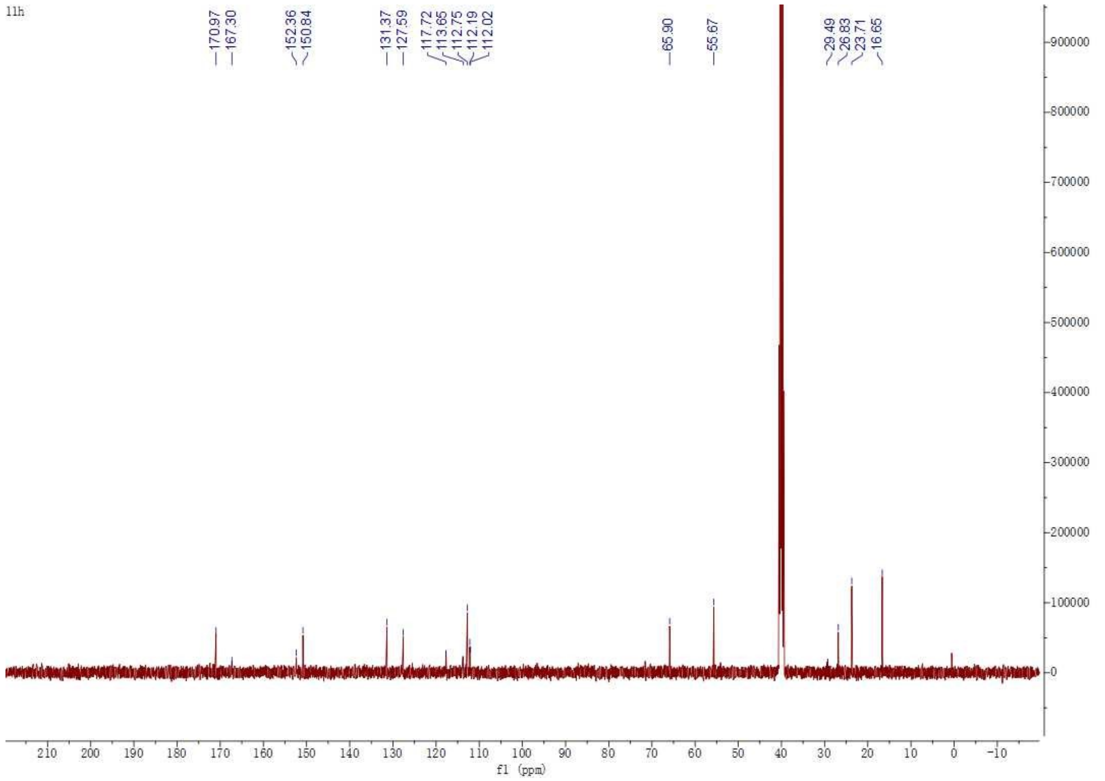

Figure S44. ^{13}C NMR spectrum **11h**.

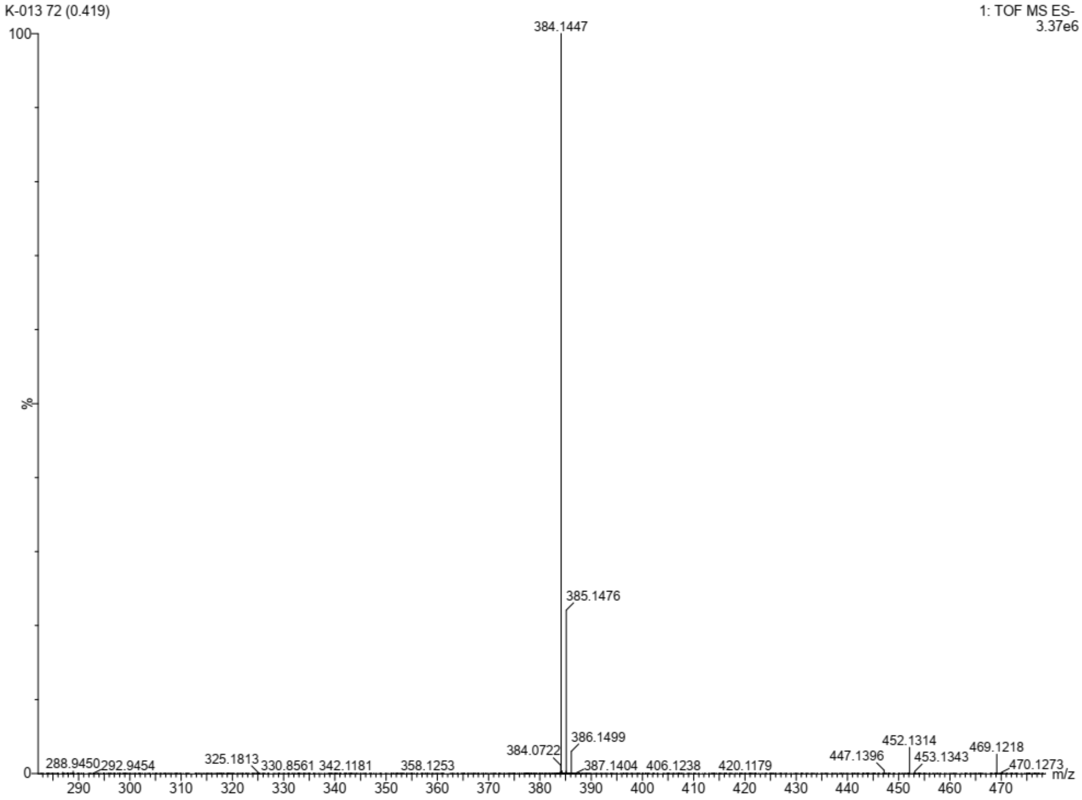

Figure S45. HRMS spectrum **11h**.
